# Microbial diversity in soils suppressive to *Fusarium* diseases

**DOI:** 10.3389/fpls.2023.1228749

**Published:** 2023-12-04

**Authors:** Irena Todorović, Yvan Moënne-Loccoz, Vera Raičević, Jelena Jovičić-Petrović, Daniel Muller

**Affiliations:** ^1^ Université Claude Bernard Lyon 1, CNRS, INRAE, VetAgro Sup, UMR5557 Ecologie Microbienne, Villeurbanne, France; ^2^ University of Belgrade, Faculty of Agriculture, Belgrade, Serbia

**Keywords:** deoxynivalenol, nivalenol, zearalenone, *Fusarium* head blight, induced systemic resistance, lipopolysaccharides

## Abstract

*Fusarium* species are cosmopolitan soil phytopathogens from the division *Ascomycota*, which produce mycotoxins and cause significant economic losses of crop plants. However, soils suppressive to *Fusarium* diseases are known to occur, and recent knowledge on microbial diversity in these soils has shed new lights on phytoprotection effects. In this review, we synthesize current knowledge on soils suppressive to *Fusarium* diseases and the role of their rhizosphere microbiota in phytoprotection. This is an important issue, as disease does not develop significantly in suppressive soils even though pathogenic *Fusarium* and susceptible host plant are present, and weather conditions are suitable for disease. Soils suppressive to *Fusarium* diseases are documented in different regions of the world. They contain biocontrol microorganisms, which act by inducing plants’ resistance to the pathogen, competing with or inhibiting the pathogen, or parasitizing the pathogen. In particular, some of the *Bacillus*, *Pseudomonas*, *Paenibacillus* and *Streptomyces* species are involved in plant protection from *Fusarium* diseases. Besides specific bacterial populations involved in disease suppression, next-generation sequencing and ecological networks have largely contributed to the understanding of microbial communities in soils suppressive or not to *Fusarium* diseases, revealing different microbial community patterns and differences for a notable number of taxa, according to the *Fusarium* pathosystem, the host plant and the origin of the soil. Agricultural practices can significantly influence soil suppressiveness to *Fusarium* diseases by influencing soil microbiota ecology. Research on microbial modes of action and diversity in suppressive soils should help guide the development of effective farming practices for *Fusarium* disease management in sustainable agriculture.

## 1 Introduction

The fungal genus *Fusarium* encompasses several plant-pathogenic species, which are among the most destructive phytopathogens world-wide, causing diseases on many agricultural crops (Burgess and Bryden, 2012). They are ubiquitous in parts of the world where cereals and other crops are grown and they produce a wide variety of mycotoxins, which may be present in feed and food products (Babadoost, 2018; Moretti et al., 2018; Chen et al., 2019). Consumption of products that are contaminated with mycotoxins may cause acute or chronic effects in both animals and humans, and could result in immune-suppressive or carcinogenic effects (Jard et al., 2011). By producing mycotoxins and by inducing necrosis and wilting in plants, *Fusarium* fungi are causing huge economic losses of cereal crops throughout the world (Khan et al., 2017). Their broad distribution has been attributed to their ability to develop on different substrates and plant species, and to produce spores that enable efficient propagation (Desjardins, 2006; Arie, 2019). They are typical soil-borne microorganisms, routinely found in plant-associated fungal communities (Reyes Gaige et al., 2020).

Efficient management of plant diseases caused by *Fusarium* is important to limit crop losses and to reduce mycotoxin production in alimentary products (Babadoost, 2018). Because mycotoxin synthesis can occur not only after harvesting but also before, one of the best ways to reduce its presence in food and feed products is to prevent its formation in the crop (Jard et al., 2011). Over the years, different methods, such as the use of resistant cultivars and chemical fungicides, have been undertaken in order to control or prevent crop diseases (Willocquet et al., 2021). In spite of that, *Fusarium* continues to cause huge crop losses, up to 70% in South America, 54% in the United States and 50% in Europe in the case of Fusarium head blight (FHB) disease of wheat (Scott et al., 2021).

Alternative control methods, based on plant-protection effects of beneficial microorganisms, have also been investigated (Janvier et al., 2007; Nguyen et al., 2018). Farming practices greatly influence these effects by shaping the rhizosphere microbial community (Campos et al., 2016), stimulating the activity of beneficial rhizosphere microorganisms and restricting the activity of soil-borne *Fusarium* pathogens (Janvier et al., 2007). Indeed, crop rotation, tillage and addition of organic amendments may provide some control of soil-borne pathogens, through different microbial direct and indirect mechanisms (Janvier et al., 2007). The effect of plant-protecting soil microbiota on plant health is of particular interest in the case of disease-suppressive soils, which were defined by Baker and Cook (1974) as “soils in which the pathogen does not establish or persist, establishes but causes little or no damage, or establishes and causes disease for a while but thereafter the disease is less important, although the pathogen may persist in the soil”. Suppressive soils represent a reservoir of beneficial microorganisms, which may confer effective plant protection against various soil-borne diseases (Gómez Expósito et al., 2017). This biocontrol potential of suppressive soils is of great importance when considering phytopathogens like *Fusarium* spp., which are causing increasing damage to crops in the on-going climate change context (Babadoost, 2018). Insight into the time and space microbial dynamics of soils suppressive to *Fusarium* diseases, together with the understanding of microbial modes of action and agricultural practices applied, is needed in order to develop safe, effective, and stable tools for disease management (Gómez Expósito et al., 2017).

By selecting their rhizosphere microbiome (Tkacz et al., 2015; Gruet et al., 2023), plants may contribute themselves to suppressiveness (Almario et al., 2014; Gómez Expósito et al., 2017). Soil represents the richest known reservoir of microbial biodiversity (Curtis et al., 2002; Wang et al., 2016) and displays several compartments, i.e. the bulk soil containing microorganisms that are not affected by the roots, the rhizosphere where soil microorganisms are under the influence of roots (and roots exudates), the rhizoplane with root-adhering microorganisms, and the endosphere for root tissues colonized by microorganisms (Sánchez-Cañizares et al., 2017). The rhizosphere and rhizoplane harbor an abundant community of bacteria, archaea, oomycetes and fungi, whose individual members can have beneficial, deleterious or neutral effects on the plant. The collective genome of this microbial community is larger than that of the plant itself, and is often referred to as the plant’s second genome (Berendsen et al., 2012). Thus, this alliance of the plant and its associated microorganisms represents a holobiont, which has interdependent, fine-tuned and complex functioning (Berendsen et al., 2012; Vandenkoornhuyse et al., 2015; Sánchez-Cañizares et al., 2017). In this system, the plant is a key player, as nearly 40% of all photosynthates are released directly by roots into the rhizosphere, serving as a fuel for microbial communities, thus recruiting and shaping this microbiome (Berendsen et al., 2012; Tkacz and Poole, 2015). These photosynthates are conditioned by the plant genotype, developmental stage, metabolism, immune system and its ability to exudate (Sánchez-Cañizares et al., 2017). In this context, suppressiveness will depend on microbiome diversity and functioning.

This review deals with recent knowledge on soils suppressive to *Fusarium* diseases, which sheds new lights on molecular and ecological mechanisms underpinning phytoprotection effects and highlights the importance of microbial diversity in the functioning of these suppressive soils. To this end, we summarize current knowledge on *Fusarium* taxonomy and ecology, and their mechanisms of plant infection. In addition, we review our understanding of biocontrol agents against *Fusarium* and their modes of action. Finally, we focus on soils suppressive to *Fusarium* diseases and the importance of farming and environmental factors modulating suppressiveness, with an emphasis on the particularities of the different *Fusarium* pathosystems.

## 2 *Fusarium* phytopathogens and plant diseases

### 2.1 *Fusarium* ecology

*Fusarium* species occur in soils, but they can also grow in and on living and dead plants (Laraba et al., 2021) and animals (Xia et al., 2019), with the ability to live as parasites or saprophytes (Smith, 2007; Summerell, 2019). Some can also be found in caves (Bastian et al., 2010) or in man-made water systems (Sautour et al., 2012). *Fusarium* species are mostly known as phytopathogens, but some of them have been evidenced as contaminants in industrial processes, indoor environments, or pharmaceutical and food products (Abdel-Azeem et al., 2019), whereas others behave as opportunistic human/animal pathogens (Al-Hatmi et al., 2019; da Silva Santos et al., 2020) or are fungicolous (Torbati et al., 2021).

**Figure 1:**
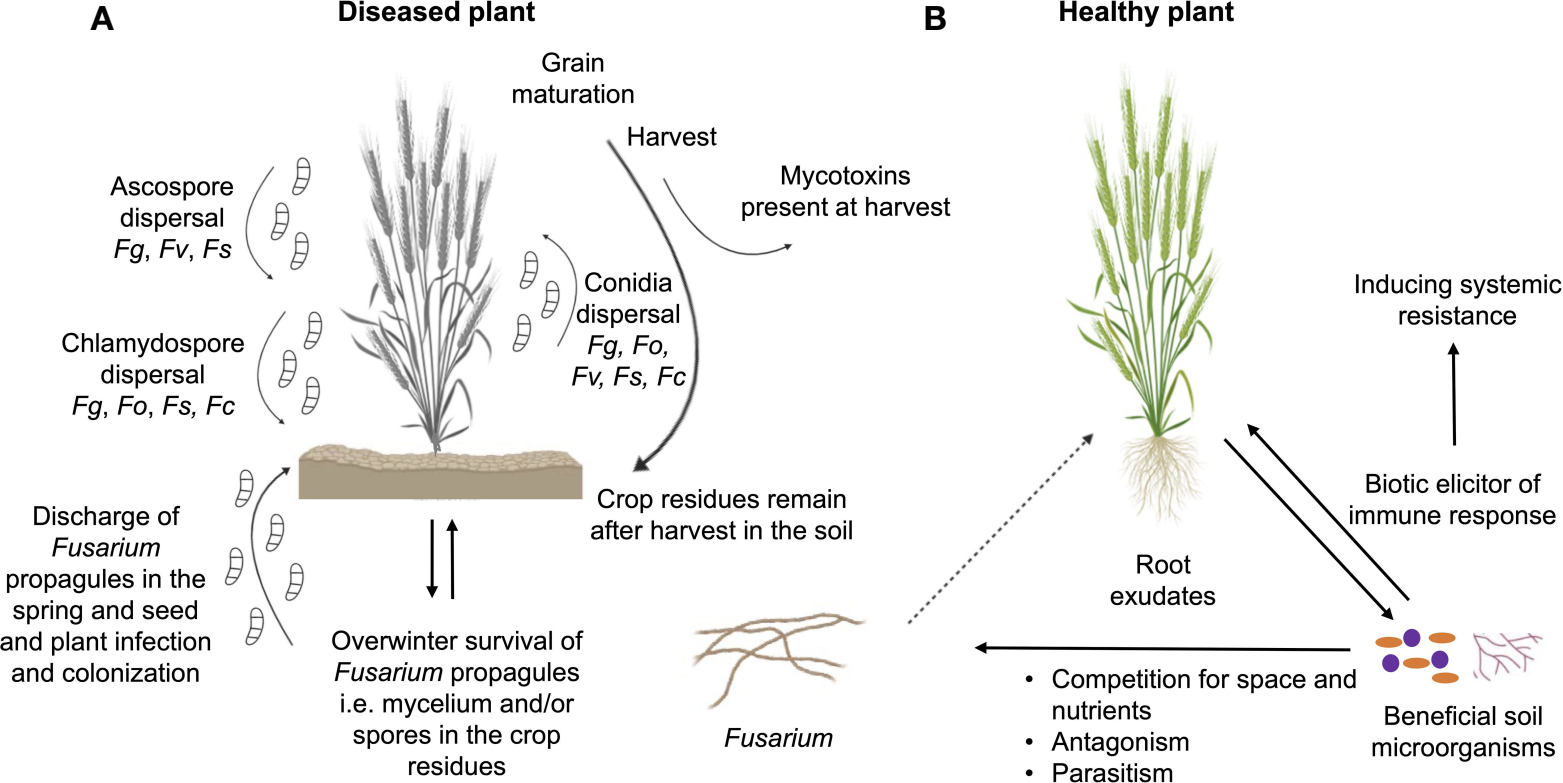
Interactions of *Fusarium* species with plant and other microbiota members. **(A)** Life cycle of *Fusarium* species and their mechanism of plant infection by producing three types of spores: ascospores, conidia and chlamydospores. *Fg*, *F graminearum*; *Fo*, *F oxysporum*; *Fs*, *F solani*; *Fc*, *F culmorum*; *Fv*, *F verticillioides*. **(B)** Dynamic interactions between beneficial soil microorganisms, plant and phytopathogenic *Fusarium* species.

Focusing on plant-interacting *Fusarium* species, their saprophytic potential enables them to survive the winter in the crop debris, in the form of mycelium or spores that serve as plant-infecting propagules in the spring (**Figure 1A**) (Leslie and Summerell, 2006). *Fusarium* species vary in reproduction strategies, and they produce sexual spores as well as three types of asexual spores, i.e. (i) microconidia, which are typically produced under all environmental conditions, (ii) macroconidia, which are often found on the surface of diseased plants, and (iii) chlamydospores (survival structures), which are thick walled and produced from macroconidia or older mycelium (Ajmal et al., 2023). More than 80% of *Fusarium* species propagate using asexual spores, but not all of them produce all three types of spores, while sexual reproduction can involve self-fertility or out-crossing (Rana et al., 2017). Additionally, some species produce sclerotia, which promote survival in soil (Leslie and Summerell, 2006).

*Fusarium* shows climatic preferences, as *F. oxysporum*, *F. solani*, *F. verticillioides* (formerly *F. moniliforme*), *F. tricinctum, F. fujikuroi, F. pseudograminearum* and *F. graminearum* are found worldwide, *F. culmorum and F. avenaceum* in temperate regions, whereas some species occur in tropical or cool regions (Backhouse and Burgess, 2002; Babadoost, 2018; Senatore et al., 2021). The growth of each *Fusarium* species is largely determined by abiotic environmental conditions, notably temperature and humidity (**Table S1**) (Xu, 2003; Crous et al., 2021). However, other environmental factors, such as soil characteristics, cropping systems, agricultural practices and other human activities may influence the diversity of *Fusarium* in soils (Abdel-Azeem et al., 2019; Pfordt et al., 2020; Wang et al., 2020; Du et al., 2022).

### 2.2 Taxonomy of *Fusarium* spp.

The *Fusarium* genus exhibits high level of variability in terms of morphological, physiological and ecological properties, which represents a difficulty in establishing a consistent taxonomy of these species (Burgess et al., 1996). An additional difficulty for classification is the existence of both asexual (anamorph) and sexual (teleomorph) phases in their life cycle (Summerell, 2019). Based on the most widely used classification, the anamorph state of the genus *Fusarium* is classified in the family *Nectriaceae*, order *Hypocreales* and division *Ascomycota* (Crous et al., 2021). Several teleomorphs have been related to *Fusarium* species, but not all *Fusarium* species have a known sexual state in their life cycle (Munkvold, 2017). Most of these teleomorphs are in the genus *Gibberella*, including the economically important pathogens, such as *G. zeae* (anamorph *F. verticillioides*) and *G. moniliformis* (anamorph *F. verticillioides*) (Keszthelyi et al., 2007). Other *Fusarium* teleomorphs are members of the genera *Albonectria*, *Neocosmospora* or *Haematonectria*. Teleomorphs are usually not observed in the field, but rather under lab conditions. The dual anamorph-teleomorph nomenclature for fungi has now been abolished, and the name *Fusarium* has been retained for these fungi (Geiser et al., 2013).

**Figure 2:**
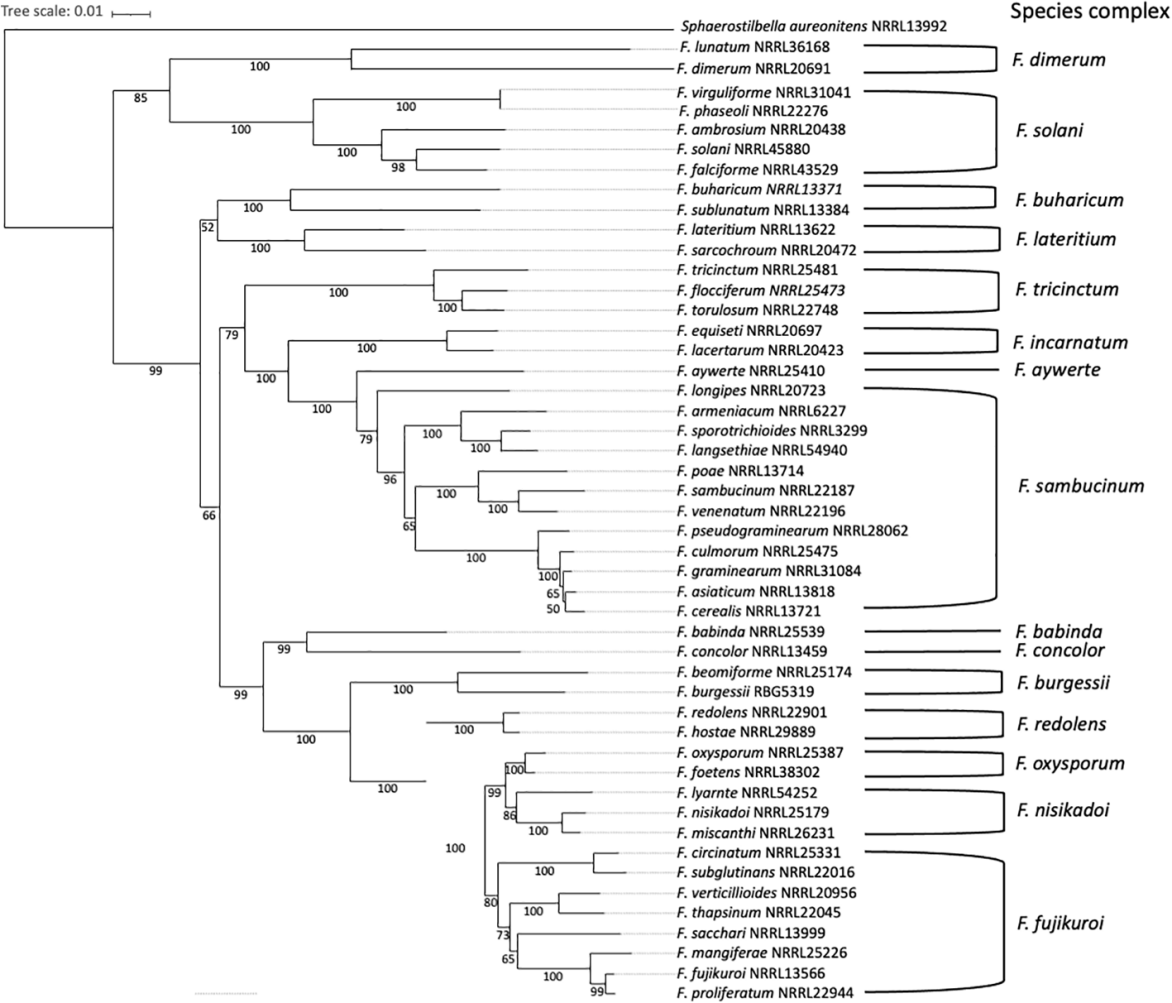
Phylogenetic relationship between pathogenic *Fusarium* species and 15 different species complexes. The distance-method tree (1000 bootstrap replicates) was inferred from the *rpb1* (RNA Polymerase 1) data set, using the SeaView multiplatform (Gouy et al., 2010). The tree was visualized using iTol (Letunic and Bork, 2021). *Sphaerostilbella aureonitens* NRRL 13992 was used as an outgroup. Species complexes delimitation is based on the phylogeny published in Summerell (2019).

The genus *Fusarium* is currently composed of 23 species complexes and at least 69 well-individualized species. *Fusarium* species complexes are groups of closely-related species with the same morphology, which are strongly supported from a phylogenetic perspective (O’Donnell et al., 2013; O’Donnell et al., 2015; Summerell, 2019; Xia et al., 2019; Laraba et al., 2021; Senatore et al., 2021; Yilmaz et al., 2021), as shown in **Figure 2**. Within a given *Fusarium* species, certain strains may be pathogenic while others are not (Fuchs et al., 1997; De Lamo and Takken, 2020; Constantin et al., 2021). However, most phytopathogenic species belong to the *F. fujikuroi*, *F. sambucinum*, *F. oxysporum, F. tricinctum* or *F. solani* species complexes (O’Donnell et al., 2013; Senatore et al., 2021). Furthermore, *Fusarium* species capable of infecting a wide range of plants are classified into different *formae speciales*, based on the host plant they can infect (Edel-Hermann and Lecomte, 2019; Coleman, 2016). Currently, there are 106 well-described *F. oxysporum formae speciales* (Edel-Hermann and Lecomte, 2019) and 12 well-described *F. solani formae speciales* (Šišić et al., 2018).

Over the past 100 years, the taxonomy of *Fusarium* has undergone many changes, but most classification procedures have been based on the size and shape of the macroconidia, the presence or absence of microconidia and chlamydospores, and the structure of the conidiophores (Ristić, 2012). Identification of *Fusarium* species based on morphological characteristics also included observations of colony pigmentation and type of aerial mycelium (Crous et al., 2021). The standard method now used to identify *Fusarium* isolates to a species level is to sequence one (or more) of the following genes: translocation elongation factor-1α (*tef-1α*), RNA polymerase 1 and 2 (*rpb1* and *rpb2*), β-tubulin (*tub*), histone (*his*), ATP citrate lyase (*acl1*) or calmodulin (*CaM*) (Herron et al., 2015; Summerell, 2019; Crous et al., 2021; Laraba et al., 2021; Yilmaz et al., 2021). The *tef-1α* gene is a first-choice marker as it has good resolution power for the majority of *Fusarium* species, while sequencing the gene *rpb2* allows differentiation of close species. The other genetic markers mentioned have variable resolution power and are often used together with *tef-1α* or *rpb2* (Crous et al., 2021). The internal transcribed spacer regions of the ribosomal gene (*ITS*), which are common barcodes to identify fungi, are not recommended for *Fusarium* identification, as they are not sufficiently informative for a significant number of *Fusarium* species (Summerell, 2019).

### 2.3 Mechanisms of *Fusarium* infection, symptoms and etiology

Before infecting the host plant tissues, soil-borne pathogens may grow in the rhizosphere or on the host as saprophytes, managing to escape the rhizosphere battlefield (Raaijmakers et al., 2009). The outcome is directly influenced by host and microbial defense mechanisms, at the level of the holobiont (Berendsen et al., 2012; Vandenkoornhuyse et al., 2015). During their life cycle, plants are exposed to numerous phytopathogens, and they have developed different adaptive strategies. Upon pathogen attack, both composition and quantity of root metabolites may change (Rolfe et al., 2019), which can be useful for direct defense against the pathogens (Rizaludin et al., 2021), for signaling the impending threat to the neighboring plants (Pélissier et al., 2021), or for recruiting beneficial microorganisms with biocontrol capabilities. The latter phenomenon is referred to as the ‘a cry for help’ strategy (Rizaludin et al., 2021).

If the pathogen manages to escape from the rhizosphere battlefield, the infection cycle can proceed. Plant infection by *Fusarium* occurs in a few successive stages (**Figure 1A**), which differs according to *Fusarium* species. Seeds infected with *Fusarium* in the previous season can also serve as disease initiators (Jiménez-Díaz et al., 2015). *F. graminearum* grows saprophytically on crop debris, which is the overwintering reservoir of the pathogen (Brown et al., 2010). The fungus may infect roots and cause damage to the collar (Ares et al., 2004). During the crop anthesis and under warm and humid weather conditions, asexual conidia, sexual ascospores or chlamydospores are dispersed by rain or wind and reach the outer anthers and outer glumes of the plant. After spore germination, hyphae penetrate the host plant through the cracked anthers, followed by inter- and intracellular mycelial growth, resulting in damage to host tissues and especially head blight disease (Brown et al., 2010). Unlike *F. graminearum*, *F. culmorum* produces only asexual conidia and chlamydospores, which are also dispersed by rain and wind, reaching plant heads and infecting the ears during the anthesis. Subsequently, conidia germinate on the lemma and palea, followed by inter- and intracellular mycelial growth (Wagacha and Muthomi, 2007). In contrast, the infection cycle of *F. oxysporum* begins when mycelia, germinating asexual conidia or chlamydspores enter the healthy plant through the root tip, lateral roots or root wounds. The fungus progresses intracellularly, entering the xylem sap flow and being transported to the aerial parts of the plant where it forms infection structures. The infection structures that form close the vascular vessels, disrupt nutrient translocation, leading to stomatal closure, leaf wilting and plant death (Banerjee and Mittra, 2018; Redkar et al., 2022a; Redkar et al., 2022b). In the case of *F. verticillioides*, infection starts when mycelia, asexual conidia or sexual ascospores are carried inside the seed or on the seed surface and later develop inside the growing plant, moving from the roots up to the maize kernels (Oren et al., 2003; Gai et al., 2018). Sometimes, the fungus colonizes and grows along the veins of the plant root, while sometimes it manages to penetrate the plant cells and form internal hyphae, therefore causing damage (Lei et al., 2011; Blacutt et al., 2018). Finally, for *F. solani*, the attachment of mycelia, asexual conidia, sexual ascospores or chlamydospores to the susceptible host is the first step in disease development, after which the fungus enters the host through stomata or the epidermis. Following penetration, *F. solani* is able to spread through the xylem, ultimately causing wilting of the host plant (Coleman, 2016).

It is reported that mycotoxins play a key role in pathogenesis, and that the aggressiveness of *Fusarium* depends on its toxin-producing capacity (Mesterházy, 2002; Xia et al., 2019; Laraba et al., 2021; Senatore et al., 2021; Yilmaz et al., 2021). Several mycotoxins are produced by *Fusarium* species, including the trichothecenes deoxynivalenol (DON) and nivalenol (NIV), zearalenone (ZEA), the cyclodepsipeptides beauvericin (BEA) and enniatins (ENN), and fusaric acid (Wagacha and Muthomi, 2007; Munkvold et al., 2021). The biosynthesis of these toxins is encoded by the *tri*, *pks, bea* and *fus* genes, respectively (Dhanti et al., 2017). However, not every species has the ability of producing all of the abovementioned mycotoxins. For example, DON and NIV are commonly produced by *F. graminearum* and *F. culmorum*, while ZEA and fusaric acid are often produced by some members of the *F. sambucinum* species complex (i.e. *F. graminearum*, *F. culmorum*), the *F. fujikuroi* complex (*F. verticillioides*) and the *F. incarnatum-equiseti* complex (Nešić et al., 2014; Munkvold et al., 2021), and BEA and ENN are produced by certain *F. oxysporum* and members of the *F. tricinctum* species complex (Munkvold et al., 2021; Senatore et al., 2021). DON production by *F. graminearum* is reported to be essential for disease development in wheat spikes (Cuzick et al., 2008). Spikes treated with DON or NIV led to yield losses even in the absence of the pathogen, indicating a strong negative effect of these trichothecenes on wheat growth (Ittu et al., 1995). In addition to DON, fusaric acid is also a virulence factor involved in programmed cell death (López-Díaz et al., 2018). It was shown that alkaline pH and low nitrogen and iron availabilities lead to increased fusaric acid production in *F. oxysporum* (Palmieri et al., 2023). Besides mycotoxins, there are other metabolites produced by *Fusarium* species that play a role in disease pathogenesis. Deletion of the *F. graminearum* gene cluster responsible for the synthesis of fusaoctaxin A abolished the fungal ability to colonize wheat coleoptiles (Jia et al., 2019). Extracellular lipases secreted by *F. graminearum* affected the plant’s defense responses by inhibiting callose synthase activity (Blümke et al., 2014).

Diseases caused by *Fusarium* species include blights, wilts and rots of various crops in natural environments and in agroecosystems (Nelson et al., 1994; Ma et al., 2013). Fusarium Head Blight (FHB) or ‘scab’ is a disease caused primarily by the *F. graminearum* species complex. It is the fourth-ranked fungal phytopathogen in term of economic importance (Dean et al., 2012; Legrand et al., 2017), causing yield losses of 20% to 70% (Bai and Shaner, 1994). *F. graminearum* is responsible for kernel damage and mycotoxin production (Ma et al., 2013) in cereals like wheat, barley, rice and oats (Goswami and Kistler, 2004). Typical symptoms of FHB begin soon after flowering, as diseased spikelets gradually bleach, leading to bleaching of the entire head. After this stage, black spherical structures called perithecia may appear on the surface of diseased spikelets. Later, as the disease becomes more severe, the fungus begins to attack the kernels inside the head, causing them to wrinkle and shrink (Schmale and Bergstrom, 2003). FHB can also be caused by *F. culmorum*, which is dominant in cooler regions of Europe (Wagacha and Muthomi, 2007). Vascular wilt is responsible for severe losses in crops such as melon, tomato, cotton, bean and banana. It is caused by *Fusarium oxysporum*, the fifth most economically important fungal phytopathogen (Michielse and Rep, 2009; Dean et al., 2012; Husaini et al., 2018). Symptoms of vascular wilt are first observed on the older leaves, as they begin to droop, followed by defoliation and yellowing of the younger leaves and eventually, plant death (Britannica, 2017; Redkar et al., 2022a). Root, stem and foot rots of various non-grain host plants are often caused by *Fusarium solani*, and the disease symptoms depend on the host plant and the particular *forma specialis* (Voigt, 2002; Coleman, 2016). However, typical symptoms of root, stem and foot rots include brown lesions on the affected plant organs. *Fusarium verticillioides* causes ear and stalk rot in hosts such as maize, sorghum and rice (Murillo-Williams and Munkvold, 2008; Dastjerdi and Karlovsky, 2015), whereas *F. graminearum* is responsible for causing *Fusarium* ear and stalk rot in maize (Goswami and Kistler, 2004). *Fusarium* ear rot is characterized by discoloration of single or multiple kernels in different areas of the ear, while early signs of stalk rot include lodging and discoloration of the stem.

## 3 Biocontrol agents against *Fusarium* and their modes of action

Plant-beneficial microorganisms present in the rhizosphere may protect plants from *Fusarium* pathogens, through different modes of action including (i) induction of resistance in the plant, (ii) competition with the pathogens for space and nutrients, (iii) amensalism based on the production of different metabolites or lytic enzymes, or (iv) parasitism (**Figure 1B**) (Nguvo and Gao, 2019; Morimura et al., 2020). Some of them are also able to inhibit mycotoxin synthesis or to enhance their detoxification (Legrand et al., 2017; Morimura et al., 2020). Certain biocontrol microorganisms have multiple modes of action, which may be expressed simultaneously or sequentially (Legrand et al., 2017).

### 3.1 Induced systemic resistance

Induced Systemic Resistance (ISR) is the phenomenon whereby a plant, once appropriately stimulated by biological or chemical inducers, exhibits enhanced resistance when challenged by a pathogen (Walters et al., 2013). ISR involves (i) the plant perception of inducing signals, (ii) signal transduction by plant tissues, and (iii) expression of plant mechanisms inhibiting penetration of the pathogen into the host tissues (Magotra et al., 2016). A wide variety of microorganisms, including the bacteria *Pseudomonas, Bacillus, Streptomyces* and the fungi *Trichoderma* and non-pathogenic *F. oxysporum* can induce ISR (Fuchs et al., 1997; Choudhary et al., 2007; Zhao et al., 2014; Galletti et al., 2020) in plants against *Fusarium* (**Table 1**). ISR in the plant-*Fusarium* system is based on microbial induction of the activity of various defense-related enzymes in plants, such as chitinase (Amer et al., 2014), lipoxygenase (Aydi Ben Abdallah et al., 2017), polyphenol oxidase (Akram et al., 2013), peroxidase, phenylalanine ammonia-lyase (Zhao et al., 2012), β-1,3-glucanase, catalase (Sundaramoorthy et al., 2012), and also the accumulation of phytoalexins, defense metabolites against fungi (Kuć, 1995). Cyclic lipopeptide antibiotics, e.g. fusaricidin (Li and Chen, 2019) and external cell components, e.g. lipopolysaccharides (LPS) (Leeman et al., 1995) can also trigger ISR. Some biocontrol agents can lead to ISR in different plant species, while other biocontrol agents show plant species specificity, suggesting specific recognition between microorganisms and receptors on the root surface (Choudhary et al., 2007).

**Table 1:** Biocontrol agents, plant-*Fusarium* systems and ISR mechanisms.

Biocontrol agent	Plant	Pathogen	Mechanism	Reference
*Bacillus amyloliquefaciens*	Tomato	*F. oxysporum*	Induction of genes coding for lipoxygenase or pathogenesis-related (PR) proteins, i.e. acidic protein PR-1 and PR-3 chitinases	Aydi Ben Abdallah et al., 2017
*Bacillus thuringiensis*	Tomato	*F. oxysporum*	Increase in polyphenol oxidase, phenyl ammonia lyase and peroxidase in plant	Akram et al., 2013
*Bacillus megaterium*	Tomato	*F. oxysporum*	Induction of chitinase, β-1,3-glucanase, peroxidase and polyphenol oxidase activities in plant	Amer et al., 2014
*Bacillus subtilis*	Tomato	*F. oxysporum*	Increased activities of phenylalanine ammonia-lyase, polyphenol oxidase, and peroxidase enzymes in plant	Akram et al., 2015
*Bacillus subtilis* and *Pseudomonas protegens* (in combination and alone)	Chilli	*F. solani*	Increased activities of peroxidase, polyphenol oxidase, phenylalanine ammonia lyase, β-1,3-glucanase, chitinase enzymes and phenol compounds involved in the synthesis of phytoalexins	Sundaramoorthy et al., 2012
*Bacillus* sp., *Brevibacillus brevis* and *Mesorhizobium ciceri* (in combination)	Chickpea	*F. oxysporum*	Increase in peroxidase, polyphenol oxidase, phenylalanine ammonia lyase, phenols and total proteins in plants	Kumari and Khanna, 2019
*Brevibacillus parabrevis*	Cumin	*F. oxysporum*	Increase in peroxidase and polyphenol oxidase in plants	Abo-Elyousr et al., 2022
*Burkholderia gladioli*	Saffron	*F. oxysporum*	Increased levels of endogenous jasmonic acid (JA) and expression of JA-regulated and plant defense genes	Ahmad et al., 2022
*Pseudomonas aeruginosa*	Tomato	*F. oxysporum*	Bacterial production of 3-hydroxy-5-methoxy benzene methanol	Fatima and Anjum, 2017
*Pseudomonas simiae*	Tomato	*F. oxysporum*	Bacterial production of lipopolysaccharides	Duijff et al., 1997
*Pseudomonas defensor*	Radish	*F. oxysporum*	Bacterial production of lipopolysaccharides	Leeman et al., 1995
*Paenibacillus polymyxa*	Cucumber	*F. oxysporum*	Bacterial production of fusaricidin, which induces ISR via salicylic acid	Li and Chen, 2019
*P. fluorescens*	Barley	*F. culmorum*	Changed transcript levels of lipid transfer proteins and protease inhibitors	Petti et al., 2010
*Streptomyces enissocaesilis*	Tomato	*F. oxysporum*	Increased catalase activity in plant	Abbasi et al., 2019
*Streptomyces rochei*	Tomato	*F. oxysporum*	Increased catalase and peroxidase activity in plant	Abbasi et al., 2019
*Streptomyces bikiniensis*	Cucumber	*F. oxysporum*	Increased activities of peroxidase, phenylalanine ammonia-lyase, and β-1,3-glucanase in plant	Zhao et al., 2012
*Trichoderma gamsii*	Maize	*F. verticillioides*	Enhanced transcript levels of ISR marker genes	Galletti et al., 2020
*Trichoderma longibrachiatum*	Onion	*F. oxysporum*	Accumulation of 25 stress-response metabolites	Abdelrahman et al., 2016
Non-pathogenic *Fusarium oxysporum*	Tomato	*F. oxysporum*	Increased activities of chitinase, β-1,3-glucanase and β-1,4-glucosidase	Fuchs et al., 1997

*Bacillus amyloliquefaciens* subsp. *plantarum* strain SV65 was assessed on tomato plants infected or not with *F. oxysporum* f. sp. *lycopersici* (FOL). The expression of genes coding for lipoxygenase or pathogenesis-related (PR) proteins, i.e. acidic protein PR-1 and PR-3 chitinases was induced by *B. amyloliquefaciens* subsp. *plantarum* SV65 in both FOL-inoculated and uninoculated plants, suggesting its ability to induce ISR (Aydi Ben Abdallah et al., 2017). Inoculation of chilli plants with *Bacillus subtilis* EPCO16 and EPC5 and *P. protegens* Pf1, separately or in combination, induced ISR, with enhanced phytoalexin activities, and protected plants against *F. solani* (Sundaramoorthy et al., 2012). Inoculation of chickpea plants with a combination of *Bacillus* sp., *Brevibacillus brevis* and *Mesorhizobium ciceri* lead to the accumulation of peroxidase, polyphenol oxidase, phenylalanine ammonia lyase and phenols in plants as well as resistance to *F. oxysporum* (Kumari and Khanna, 2019). *Paenibacillus polymyxa* WLY78 controls Fusarium wilt, caused by *Fusarium oxysporum* f. sp. *cucumerinum*, through the production of fusaricidin, which can induce ISR in cucumber via the salicylic acid pathway (Li and Chen, 2019). Tomato showed increased catalase and peroxidase activities when treated with either *Streptomyces* sp. IC10 and Y28, or with Y28 alone, respectively, outlining a strain-specific ISR in tomato against Fusarium wilt mediated by FOL (Abbasi et al., 2019). *Streptomyces bikiniensis* increased the activities of peroxidase, phenylalanine ammonia-lyase and β-1,3-glucanase in cucumber leaves (Zhao et al., 2012). Nonpathogenic *Fusarium oxysporum* Fo47 can triger induced resistance to FOL and protects tomato from Fusarium wilt (Fuchs et al., 1999). *Trichoderma gamsii* IMO5 increased transcript levels of ISR-marker genes *ZmLOX10*, *ZmAOS* and *ZmHPL* in maize leaves, thereby protecting the plant from the pink ear rot pathogen *F. verticillioides* (Galletti et al., 2020).

An important determinant of biocontrol efficacy is the population density of ISR-triggering microorganisms. For example, ~10^5^ CFU of *Pseudomonas defensor* (ex *fluorescens*) WCS374 per g of root are required for significant suppression of Fusarium wilt of radish (Raaijmakers et al., 1995). Another important feature of ISR in plants is that its effects are not only expressed at the site of induction but also in plant parts that are distant from the site of induction (Pieterse et al., 2014). For example, root-colonizing *Pseudomonas simiae* (ex *fluorescens*) WCS417r induced resistance in carnation, with phytoalexin accumulation in stems, and protected shoots from *F. oxysporum* (Van Peer et al., 1991). Priming of barley heads with *P. fluorescens* MKB158 led to changes in the levels of 1203 transcripts (including some involved in host defense responses), upon inoculation with pathogenic *F. culmorum* (Petti et al., 2010).

### 3.2 Competition for space and nutrients

In the case of competition, biocontrol of pathogens occurs when another microorganism is able to colonize the environment faster and use nutrient sources more efficiently than the pathogen itself, especially under limited conditions (Maheshwari et al., 2013; Legrand et al., 2017). Bacteria and fungi have the ability to compete with *Fusarium*, but the underlying mechanism of competition is sometimes unclear. For example, competition between non-pathogenic *F. oxysporum* strains and pathogenic *F. oxysporum* has been described, reducing disease incidence (Eparvier and Alabouvette, 1994; Fuchs et al., 1999). Similarly, a non-aflatoxigenic *Aspergillus flavus* strain was found to outcompete a mycotoxin-producing *F. verticillioides* during colonization of maize (Reis et al., 2020). Competition may involve bacteria such as *Pseudomonas capeferrum* (ex *putida*) strain WCS358, which suppresses Fusarium wilt of radish (Lemanceau et al., 1993).

In some cases, traits involved in competition have been identified. In *P. putida* (Trevisan) Migula isolate Corvallis, competition for root colonization entails plant’s production of agglutinin, and *P. putida* mutants lacking the ability to agglutinate with this plant glycoprotein showed reduced levels of rhizosphere colonization and suppression of Fusarium wilt of cucumber (Tari and Anderson, 1988). *P. capeferrum* WCS358 suppresses Fusarium wilt of radish by competing for iron through the production of its pseudobactin siderophore (Lemanceau et al., 1993). In addition to bacteria, the fungus *Trichoderma asperellum* strain T34 can control the disease caused by *F. oxysporum* f. sp. *lycopersici* on tomato plants by competing for iron (Segarra et al., 2010).

### 3.3 Amensalism based on antibiosis or lytic enzymes

Another important microbial mechanism to suppress plant pathogens is the secretion of inhibitors by beneficial microorganisms. They include anti-fungal secondary metabolites, sometimes termed antibiotics (e.g. fengycin, iturin, surfactin (Chen et al., 2018), fusaricidin and polymyxin (Zalila-Kolsi et al., 2016)), as well as Volatile Organic Compounds (VOCs; Zaim et al., 2016; Legrand et al., 2017) (**Table 2**). Extracellular lytic enzymes such as cellulase, chitinase, pectinase, xylanase (Khan et al., 2018), protease and glucanase (Saravanakumar et al., 2017), can also interfere with *Fusarium* growth or activity.

**Table 2:** Biocontrol agents, plant-*Fusarium* systems and biocontrol enzymes and metabolites.

Biocontrol agents	*Fusarium* pathogens	Biocontrol enzymes and metabolites	References
*Bacillus subtilis*	*F. oxysporum* *F. graminearum*	Cellulase, chitinase, pectinase, xylanase, protease, fengycins and surfactins	Zhao et al., 2014; Zalila-Kolsi et al., 2016; Khan et al., 2018
*Bacillus velezensis*	*F. graminearum F. culmorum*	Fengycin B, iturin A, surfactin A and siderophores	Chen et al., 2018; Adeniji et al., 2019
*Bacillus pumilus*	*F. oxysporum*	Chitinolytic enzymes and antibiotic surfactin	Agarwal et al., 2017
*Bacillus amyloliquefaciens*	*F. graminearum*	Iturin and surfactin	Zalila-Kolsi et al., 2016
*Brevibacillus fortis*	*F. oxysporum*	Edeine	Johnson et al., 2020
*Brevibacillus reuszeri*	*F. oxysporum*	Chitinolytic enzymes	Masri et al., 2021
*Burkholderia* sp.	*F. oxysporum*	Phenazine-1-carboxylic acid	Xu et al., 2020
*Chryseobacterium* sp.	*F. solani*	VOCs	Tyc et al., 2015
*Gluconacetobacter diazotrophicus*	*F. oxysporum*	Antibiotic (pyoluteorin) and VOCs	Logeshwarn et al., 2011
*Kosakonia arachidis*	*F. verticillioides* *F. oxysporum*	Chitinase, protease, cellulase and endoglucanase	Singh et al., 2021
*Lysobacter antibioticus*	*F. graminearum*	VOCs	Kim et al., 2019
*Paenibacillus polymyxa*	*F. graminearum F. oxysporum*	Fusaricidin, polymyxin and VOCs	Raza et al., 2015; Zalila-Kolsi et al., 2016
*Pseudomonas* sp.	*F. verticillioides* *F. graminearum*	Antifungal antibiotics and fluorescent pigments	Pal et al., 2001
*Streptomyces* spp.	*F. oxysporum*	Antibiotic compounds, lipopeptin A and lipopeptin B	Cuesta et al., 2012; Wang et al., 2023
*Trichoderma* sp.	*F. oxysporum* *F. caeruleum*	Pyrones, koningins and viridins	Reino et al., 2008

*Bacillota* representatives (formerly *Firmicutes*), i.e. *Bacillus* and *Brevibacillus* species are highlighted in several studies as candidates for *Fusarium* biocontrol through production of anti-fungal metabolites (Palazzini et al., 2007; Zhao et al., 2014; Chen et al., 2018; Johnson et al., 2020). *Bacillus subtilis* SG6 has the ability to produce fengycins and surfactins acting against *F. graminearum* (Zhao et al., 2014), whereas *Bacillus velezensis* LM2303 exhibited strong inhibition of *F*. *graminearum* and significantly reduced FHB severity under field conditions (Chen et al., 2018). Genome mining of *B. velezensis* LM2303 identified 13 biosynthetic gene clusters encoding secondary metabolites and chemical analysis confirmed their presence. These metabolites included three antifungal metabolites (fengycin B, iturin A, and surfactin A) and eight antibacterial metabolites (surfactin A, butirosin, plantazolicin and hydrolyzed plantazolicin, kijanimicin, bacilysin, difficidin, bacillaene A and bacillaene B, 7-o-malonyl macrolactin A and 7-o-succinyl macrolactin A) (Chen et al., 2018). *Brevibacillus fortis* NRS-1210 produces edeine, a compound with antimicrobial activity, which inhibits chlamydospore germination and conidia growth in *F. oxysporum* f. sp. *cepae* (Johnson et al., 2020). *Pseudomonadota* representatives (formerly *Proteobacteria*) are also known for disturbing *Fusarium* growth or activity. Thin layer chromatography analysis showed that *Gluconacetobacter diazotrophicus* produces pyoluteorin, which is involved in the suppression of *F. oxysporum* (Logeshwarn et al., 2011), while *Burkholderia* sp. HQB-1 produces phenazine-1-carboxylic acid, which is efficient at controlling Fusarium wilt of banana, caused by *F. oxysporum* f. sp. *cubense* (Xu et al., 2020). *Pseudomonas* sp. EM85 was successful in suppressing disease caused by *F. verticillioides* and *F. graminearum*, by producing antifungal antibiotics and fluorescent pigments (Pal et al., 2001). Besides bacteria, *Trichoderma* fungi synthesize a number of secondary metabolites such as pyrones (which completely inhibit spore germination of *F. oxysporum*), koningins (which affect the growth of *F. oxysporum*) and viridin (which prevents the germination of spores of *F. caeruleum*) (Reino et al., 2008).

VOCs have recently received more attention, as they can enable interactions between organisms in the soil ecosystem through both water and air phases (De Boer et al., 2019). *Paenibacillus polymyxa* WR-2 produced VOCs when cultivated in the presence of organic fertilizer and root exudates. Among them, benzothiazole, benzaldehyde, undecanal, dodecanal, hexadecanal, 2-tridecanone and phenol inhibited mycelial growth and spore germination of *F. oxysporum* f. sp. *niveum* (Raza et al., 2015). *Chryseobacterium* sp. AD48 inhibited growth of *F. solani* through the production of VOCs (Tyc et al., 2015). VOCs produced by *Lysobacter antibioticus* HS124 enhanced mycelial development, but they also reduced sporulation and spore germination of *F. graminearum* at the same time (Kim et al., 2019). In addition, testing the antagonistic mechanisms of *Aspergillus pseudocaelatus* and *T. gamsii* revealed the presence of the VOCs 2,3,4-trimethoxyphenylethylamine, 3-methoxy-2-(1-methylethyl)-5-(2-methylpropyl) pyrazine, (Z)-9- octadecenamide, pyrrolo [1,2-a] pyrazine-1,4-dione, hexahydro-3-(2-methylpropyl)-, thieno [2,3-c] pyridine-3-carboxamide,4,5,6,7-tetrahydro-2-amino-6-methyl- and hexadecanamide, which have an inhibitory activity against *F. solani* (Zohair et al., 2018).

Regarding extracellular lytic enzymes, *B. subtilis* 30VD-1 inhibited FOL by producing cellulase, chitinase, pectinase, xylanase and protease (Khan et al., 2018), while *Bacillus pumilus* synthesized a chitinolytic enzyme that reduced severity of disease caused by *F. oxysporum* on buckwheat under gnotobiotic conditions (Agarwal et al., 2017). *Brevibacillus reuszeri* affected the growth of *F. oxysporum* by producing chitinolytic enzymes (Masri et al., 2021). *Kosakonia arachidis* EF1 produced different cell-wall degrading enzymes, such as chitinases, proteases, cellulases and endoglucanases, which inhibited growth of *F. verticillioides* and *F. oxysporum* f. sp. *cubense*. Scanning electron microscopy revealed broken fungal mycelia surface and hyphae fragmentation when pathogens were grown in the presence of *K. arachidis* EF1 (Singh et al., 2021).

### 3.4 Parasitism

Mycoparasitism is an ancient lifestyle, during which one fungus parasitizes another fungus (Kubicek et al., 2011). It involves direct physical contact with the host mycelium (Pal and McSpadden Gardener, 2006), secretion of cell wall-degrading enzymes and subsequent hyphal penetration (Viterbo et al., 2002). Mycoparasitic relationships can be biotrophic, where the host remains alive and the mycoparasitic fungus obtains nutrients from the mycelium of its partner, or necrotrophic, where the parasite contacts and penetrates the host, resulting in the death of the host and allowing the mycoparasite to use the remains of the host as a nutrient source (Jeffries, 1995). Several species of fungi are mycoparasitic, of which *Trichoderma* is the best described. Contact between the mycoparasitic fungi *Gliocladium roseum*, *Penicillium frequentans*, *T. atroviride*, *T. longibrachiatum* or *T. harzianum* and their phytopathogenic targets *F. culmorum*, *F. graminearum* and *F. nivale* triggers the formation of various mycoparasitic structures, such as hooks and pincers, which lead to cell disruption in the phytopathogens (Pisi et al., 2001). When *T. asperellum* and *T. harzianum* were grown in the presence of *F. solani* cell wall, they secreted several cell wall-degrading enzymes, such as β-1,3-glucanase, *N*-acetylglucosaminidases, chitinase, acid phosphatase, acid proteases and alginate lyase (Qualhato et al., 2013), and similarly, *Clonostachys rosea* produced chitinase and β-1,3-glucanase in the presence of *F. oxysporum* cell wall (Chatterton and Punja, 2009). *Sphaerodes mycoparasitica* is a biotrophic fungus that parasitizes *F. avenaceum*, *F. oxysporum* and *F. graminearum* hyphae and forms hooks as parasitic structures (Vujanović and Goh, 2009). However, the direct contribution of mycoparasitism to biological control is difficult to quantify as mycoparasitic fungi typically exhibit a number of different biocontrol mechanisms (Pal and McSpadden Gardener, 2006).

### 3.5 Inhibition and detoxification of mycotoxins

Biocontrol research often focuses on pathogen inhibition, and effects on mycotoxin synthesis or detoxification are often neglected (Pellan et al., 2020). It can be expected that *Fusarium* inhibition will diminish mycotoxin synthesis, but one comprehensive study found that *B. amyloliquefaciens* FZB42 inhibited *F. graminearum* but at the same time stimulated biosynthesis of DON toxin (Gu et al., 2017). Conversely, DON production of *F. graminearum* (on wheat kernels) was reduced by more than 80% with *B. amyloliquefaciens* WPS4-1 and WPP9 (Shi et al., 2014), and *Paenibacillus polymyxa* W1-14-3 and C1-8-b (He et al., 2009), whereas *Pseudomonas* strains MKB158 and MKB249 significantly reduced DON production in *F. culmorum*-infected wheat seeds (Khan and Doohan, 2009). *Pseudomonas* sp. MKB158 lowered expression of the gene coding for trichodiene synthase (an enzyme involved in the production of trichothecene mycotoxins in *Fusarium*) by 33%, in wheat treated with *F. culmorum* (Khan et al., 2006). DON production in both *F. graminearum* and *F. verticillioides* was also inhibited by the fungus *T. asperellum* TV1 and the oomycete *Pythium oligandrum* M1/ATCC (Pellan et al., 2020). Other mycotoxins may be targeted, as *Trichoderma harzianum* Q710613, *T. atroviride* Q710251 and *T. asperellum* Q710682 decreased ZEA production in a dual-culture assay with *F. graminearum* (Tian et al., 2018), and *Streptomyces* sp. XY006 lowered the synthesis of fusaric acid in *Fusarium oxysporum* f. sp. *cubense* (Wang et al., 2023).

## 4 Soils suppressive to *Fusarium* diseases

### 4.1 General suppressiveness

Soils that are suppressive to soil-borne diseases have been known for more than 70 years (Vasudeva and Roy, 1950), and disease suppression is associated primarily with the activity of beneficial microorganisms (Schlatter et al., 2017). These microorganisms interact with phytopathogens, thus affecting their survival, development or infection of the plant (Weller et al., 2002; Raaijmakers et al., 2009). Two types of soil suppressiveness have been described, i.e. general (microbial community-based) suppressiveness and specific (microbial population-based) suppressiveness (Schlatter et al., 2017). General suppressiveness is dependent on the entire soil microbial biomass, which causes pathogen inhibition through various mechanisms, especially competition and the microbial release of inhibitors (Garbeva et al., 2011; De Boer et al., 2019), and it cannot be transferred experimentally between the soils (Weller et al., 2002). Hence, all soils may present some level of general suppressiveness to soil-borne diseases, and this level depends on soil type, agricultural practices and total microbial activity (Janvier et al., 2007; Raaijmakers et al., 2009).

General suppressiveness typically results in the inability of the pathogen to survive and proliferate in soil, and is termed fungistasis in the case of fungal phytopathogens. Fungistasis can affect *Fusarium* pathogens (De Boer et al., 2019; Legrand et al., 2019), but its significance in relation to different *Fusarium* species or *formae speciales* needs clarification. Legrand et al. (2019) determined the soil fungistasis status of 31 wheat fields in the case of *F. graminearum*, highlighting higher bacterial diversity, a higher prevalence of *Pseudomonas* and *Bacillus* species and a denser network of co-occurring bacterial taxa in soils with fungistasis. It suggests the importance of cooperations within diversified bacterial communities (including with antagonistic taxa) to control *F. graminearum* in soil (Legrand et al., 2019). Accordingly, both bacterial and fungal communities differed between Fusarium wilt-diseased soils vs healthy (presumably suppressive) soils taken from from eight countries and grown with different crop plants (Yuan et al., 2020).

### 4.2 Specific suppressiveness to *Fusarium* diseases

Besides general suppressiveness, there is also specific suppression to certain diseases, which relies on the activity of a few plant-protecting microbial groups (Weller et al., 2007; Almario et al., 2014; Mousa and Raizada, 2016). Specific suppressiveness may be conferred to non-suppressive soils (i.e. conducive soils) by inoculating them with 0.1% - 10% of suppressive soil (Garbeva et al., 2004; Raaijmakers et al., 2009). Although abiotic factors, such as soil physicochemical properties, may contribute to the control of a given pathogen, specific suppressiveness is essentially a phenomenon mediated by beneficial soil microorganisms, since sterilization processes convert suppressive into conducive soils (Garbeva et al., 2004). It is expected that specific suppressiveness entails the contribution of a few plant-protecting microbial groups (Weller et al., 2007), but microbial community comparison of suppressive vs conducive soils may evidence significant differences for a large number of taxa (Kyselková et al., 2009; Legrand et al., 2019; Ossowicki et al., 2020; Yuan et al., 2020; Lv et al., 2023).

**Figure 3:**
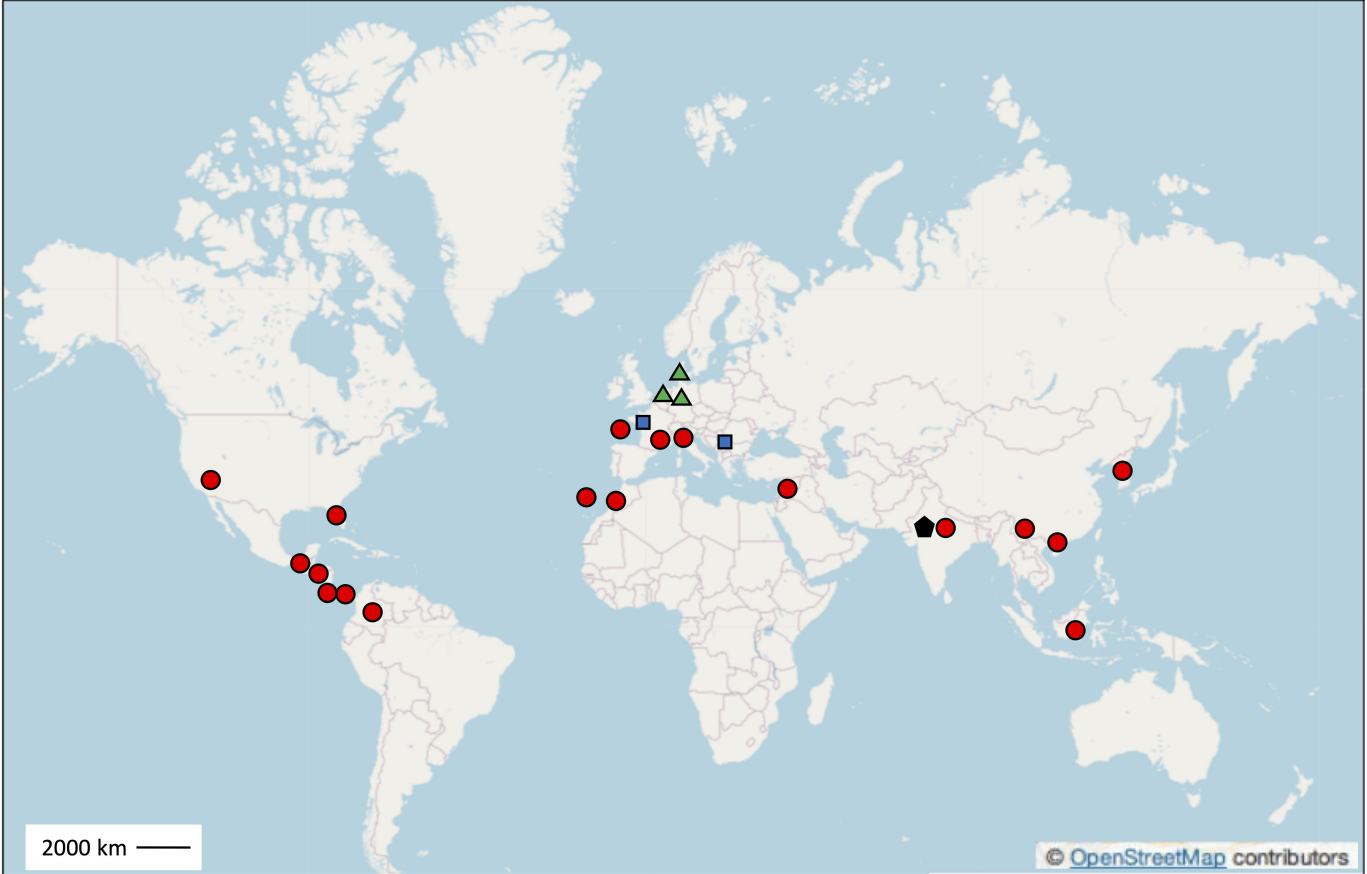
Geographic locations of the main field sites with soils documented to be suppressive to *Fusarium* diseases, in Europe including France (Noirmoutier Island, Châteaurenard in Southeast France, and Brittany), Denmark, The Netherlands, Germany, Italy (Albenga, Carmagnola, and Santena), Gran Canaria Island (Spain, located in the Atlantic Ocean), and Serbia, in North America (California and Florida), Central America (Honduras, Costa Rica, Panama, and Guatemala), South America (Colombia), Asia (Korea, China, India, Israel, and Indonesia), and Africa (Morocco). Each location is marked with the corresponding pathogen: *F. oxysporum* (indicated by a red dot), *F. culmorum* (green triangle), *F. graminearum* (blue square), and *F. udum* (black pentagon).

The phenomenon of disease suppressiveness has been described for many soil-borne fungal pathogens, including *Gaeumannomyces graminis* var. *tritici* (Shipton et al., 1973; Weller et al., 2007; Schlatter et al., 2017), *Thievalopsis basicola* (Stutz et al., 1986; Almario et al., 2014) and *Rhizoctonia solani* (Mendes et al., 2011; Schlatter et al., 2017). It is also well established in the case of several *Fusarium* pathogenic species (**Table 3**), such as *F. culmorum* on wheat (in the Netherlands and Germany; Ossowicki et al., 2020) and barley (in Denmark; Rasmussen et al., 2002), *F. oxysporum* f. sp. *albedinis* on palm tree (in Marocco; Rouxel and Sedra, 1989), *F. oxysporum* f. sp. *batatas* on sweet potato (in California; Smith and Snyder, 1971), *F. oxysporum* f. sp. *cubense* on banana (in India, Indonesia, China, Gran Canaria island and several Central America states; Stotzky and Torrence Martin, 1963; Domínguez et al., 1996; Shen et al., 2015b; Wang et al., 2019; Nisrina et al., 2021; Yadav et al., 2021; Fan et al., 2023), *F. oxysporum* f. sp. *cucumerinum* on cucumber (in California; Sneh et al., 1984) and cape gooseberry (in Colombia; Bautista et al., 2023), *F. oxysporum* f. sp. *dianthi* on carnation (in Italy; Garibaldi et al., 1983), *F. oxysporum* f. sp. *fragariae* on strawberry (in Korea; Cha et al., 2016), *F. oxysporum* f. sp. *lini* on flax (in Italy, California; Kloepper et al., 1980; Tamietti and Pramotton, 1990), *F. oxysporum* f. sp. *lycopersici* on tomato (in France, Italy; Tamietti and Alabouvette, 1986; Tamietti et al., 1993) and wheat (in Italy; Tamietti and Matta, 1984), *F. oxysporum* f. sp. *melonis* on melon (in France; Louvet et al., 1976), *F. oxysporum* f. sp. *niveum* on watermelon (in Florida; Larkin et al., 1993), *F. oxysporum* f. sp. *radicis-cucumerinum* on cucumber (in Israel; Klein et al., 2013), *F. udum* on pigeon-pea (in India; Vasudeva and Roy, 1950), and *F. graminearum* on wheat (in Serbia; Todorović et al., unpublished data). Therefore, unlike with other pathogenic taxa, suppressiveness is documented across a wide range of *Fusarium* pathosystems. It also appears that suppressiveness to *Fusarium* diseases occurs in numerous parts of the world (**Figure 3**).

**Table 3:** List of locations with soils suppressive to *Fusarium* diseases known to date, with a pathosystem, disease and the underlying suppression mechanism.

Pathogen	Disease	Country	Suppression mechanism	References
*F. culmorum*	Seedling blight of barley	Denmark	Soil microbiota that has a more efficient cellulolytic activity	Rasmussen et al., 2002
*F. culmorum*	*F. culmorum* disease in wheat	Netherlands and Germany	No specific taxa, but a guild of bacteria working together	Ossowicki et al., 2020
*F. graminearum*	No disease supression tested, only fungistasis	Britanny, France	*Pseudomonas* and *Bacillus*	Legrand et al., 2019
*F. graminearum* Fg1	Wheat damping-off	Serbia	Under progress	Todorović et al., unpublished data
*F. oxysporum* f. sp. *albedinis*	Bayoud vascular wilt of palm tree	Marocco	Competition with soil microbiota	Rouxel and Sedra, 1989
*F. oxysporum* f. sp. *melonis*	Fusarium wilt of watermelon	Châteaurenard, France	Competition with soil microbiota including non-pathogenic *Fusarium*	Louvet et al., 1976; Alabouvette et al., 1985
*F. oxysporum* f. sp. *fragariae*	Fusarium wilt of strawberry	Korea	*Streptomyces*, wilt-suppressive soil that was developed through monoculture	Cha et al., 2016
*F. oxysporum* f. sp. *dianthi*	Vascular wilting disease of carnations	Albenga, Italy	Competition with other *Fusarium*	Garibaldi et al., 1983
*F. oxysporum* f. sp. *batatas*	Fusarium wilt on sweet potato	California, USA	No data	Smith and Snyder, 1971
*F. oxysporum* f. sp. *cubense*	Fusarium wilt of banana disease	Ayodhya district, India	*Bacillus licheniformis* producing anti-fungal secondary metabolites	Yadav et al., 2021
*F. oxysporum* f. sp. *cubense*	Fusarium wilt of banana disease	Gran Canaria, Spain	Sodium in soil	Domínguez et al., 1996
*F. oxysporum* f. sp. *cubense*	Fusarium wilt of banana disease	Indonesia	*Pseudomonas* and *Burkholderia*	Nisrina et al., 2021
*F. oxysporum* f. sp. *cubense*	Fusarium wilt of banana disease	Honduras, Costa Rica, Panama and Guatemala	Clay mineralogy, presence of montmorillonite-type clay in suppressive soil	Stotzky and Torrence Martin, 1963
*F. oxysporum* f. sp. *cubense*	Fusarium wilt of banana disease	Hainan, China	*Pseudomonas* inducing jasmonate and salicylic acid pathways and shared core microbiome in suppressive soils	Shen et al., 2015b; Zhou et al., 2019; Shen et al., 2022; Lv et al., 2023; Wang et al., 2023
*F. oxysporum* f. sp. *cubense*	Fusarium wilt of banana disease	Yunnan, China	*Bacillus* and *Sphingomonas* negatively correlated to *F. oxysporum*. *B. velezensis* strain YN1910 presented biocontrol properties	Fan et al., 2023
*F. oxysporum* f. sp. *cucumerinum*	Fusarium wilt of cape gooseberry	Colombia	Higher prevalence of certain bacterial taxa	Bautista et al., 2023
*F. oxysporum* f. sp. *physalis*	Fusarium wilt of cucumber	California, USA	*Pseudomonas* siderophores and lytic bacteria	Sneh et al., 1984
*F. oxysporum* f. sp. *lini*	Fusarium wilt of flax	California, USA	*Pseudomonas* siderophores	Kloepper et al., 1980
*F. oxysporum* f. sp. *lini*	Fusarium wilt of flax	Carmagnola and Santena, Italy	Competition with other *Fusarium*	Tamietti and Pramotton, 1990
*F. oxysporum* f. sp. *lycopersici*	Fusarium wilt of tomato	Noirmoutier, France	Non-pathogenic *F. oxysporum*	Tamietti and Alabouvette, 1986
*F. oxysporum* f. sp. *lycopersici*	Fusarium wilt of wheat	Albenga, Italy	Non-pathogenic *F. oxysporum* inducing plant defense	Tamietti and Matta, 1984
*F. oxysporum* f. sp. *lycopersici*	Fusarium wilt of tomato	Albenga, Italy	Non-pathogenic *F. oxysporum* inducing plant defense	Tamietti et al., 1993
*F. oxysporum* f. sp. *niveum*	Fusarium wilt of watermelon	Florida, USA	Wilt-suppressive soil that was developed through monoculture	Larkin et al., 1993
*F. oxysporum* f. sp. *radicis- cucumerinum*	Cucumber crown and root rot	Israel	Suppressiveness induced by mixing sandy soil with wild rocket (*Diplotaxis tenuifolia*) debris under field conditions	Klein et al., 2013
*F. udum* Butl.	Wilt of pigeon-pea	Dehli, India	Soil microbiota	Vasudeva and Roy, 1950

### 4.3 Natural and induced specific suppressiveness to *Fusarium* diseases

Specific suppressiveness is sometimes an intrinsic property of the soil and persists over years, despite changing ecological conditions related to crop rotation. This natural/long-term suppressiveness is well documented for several pathosystems, for instance in Swiss soils suppressive to tobacco black root rot (*T. basicola*) near Morens (Stutz et al., 1986). Suppressive and conducive soils may be located at small geographic distances in the landscape, and differences in plant disease incidence between neighbouring fields that share similar climatic conditions and agronomic practices are attributed by the differences in the resident microbiota in these soils (Almario et al., 2014). Natural suppressiveness has also been extensively studied in the case of *Fusarium* diseases, in particular with the Fusarium wilt suppressive soils of Salinas Valley (California) or Châteaurenard (France). In these soils, Fusarium wilt disease remains minor despite the long history of cultivation of different crops, and the introduction of small amount of these soils to sterilized suppressive soil or conducive soil significantly decreased Fusarium wilt disease incidence (Scher and Baker, 1980; Alabouvette, 1986). In both locations, the small level of disease in plants cannot be attributed to the absence of *Fusarium* in the soil, but rather to plant protection by the soil microbiota (Sneh et al., 1984; Alabouvette et al., 1985; Siegel-Hertz et al., 2018), as found in later investigations (Bautista et al., 2023).

Specific disease suppressiveness can also result from particular farming practices leading to the built-up of a plant-protecting microbiota. Often, this takes place following crop monoculture, typically after early disease outbreak, and is examplified by take-all decline of wheat (Weller et al., 2002; Sanguin et al., 2009) and barley (Schreiner et al., 2010). Induced suppressiveness is initiated and maintained by monoculture, in the presence of the pathogen *Gaeumannomyces graminis* var. *tritici* (Weller et al., 2002). Soil suppressiveness to *Fusarium* diseases is usually natural, but cases of induced suppressiveness are also documented. Thus, soils found in Hainan island (China) that were grown for years with banana in confontration with pathogenic *F. oxysporum* displayed rhizosphere enrichment in microbial taxa conferring protection from banana wilt (termed banana Panama disease) (Shen et al., 2022), watermelon monoculture in Florida induced suppressiveness to wilt caused by *F. oxysporum* f. sp. *niveum* (Larkin et al., 1993), and 15 years of strawberry monoculture in Korea triggered suppressiveness to wilt caused by *F. oxysporum* f. sp. *fragariae* (Cha et al., 2016). Soil addition of wild rocket residues resulted in suppressiveness to cucumber crown and root rot (*F. oxysporum* f. sp. *radicis-cucumerinum*) in Israel (Klein et al., 2013), whereas suppressiveness to Fusarium wilt can also be induced by microbial biofertilizer inoculants reshaping the soil microbiome (Xiong et al., 2017). Thus, organic fertilizer containing *B*. *amyloliquefaciens* W19 enhanced levels of indigenous *Pseudomonas* spp. and provided suppression of Fusarium wilt of banana (Tao et al., 2020). The combined action of *B*. *amyloliquefaciens* W19 and *Pseudomonas* spp. is thought to cause a decrease in *Fusarium* density in the root zone of banana. Organic fertilizers inoculated with *Erythrobacter* sp. YH-07 controlled Fusarium wilt in tomato, as a direct result of the bacteria and indirectly by altering the composition of the microbial community (Tang et al., 2023). Organic fertilizer amended with *Bacillus* and *Trichoderma* resulted in an increase in indigenous *Lysobacter* spp., thus indirectly inducing suppression of Fusarium wilt of vanilla (Xiong et al., 2017).

## 5 The microbiome of soils suppressive to *Fusarium* diseases

### 5.1 Biocontrol microorganisms in soils suppressive to *Fusarium* diseases

Many biocontrol strains originate from suppressive soils, and they were investigated as a mean to understand disease suppressiveness. In the case of *Fusarium* diseases, examples include *Pseudomonas* sp. Q2-87 (*P. corrugata* subgroup) (Weller et al., 2007), isolated from wheat in take-all decline soils but that protects tomato from *F. oxysporum* f. sp. *radicis-lycopersici*, *Pseudomonas* sp. C7 (*P. corrugata* subgroup) (Lemanceau and Alabouvette, 1991) isolated from soil suppressive to Fusarium wilt of tomato, and non-pathogenic *F. oxysporum* strains Fo47 (Fuchs et al., 1997; Duijff et al., 1998; Fuchs et al., 1999), CAV 255 (Sajeena et al., 2020) and Ro-3 (Bubici et al., 2019). Based on the biocontrol traits thus identified, the corresponding microbial functional groups have been characterized in suppressive vs conducive soils, using isolate collections, molecular fingerprints or sequencing. Fluorescent *Pseudomonas* bacteria, especially those producing the antifungal metabolite 2,4-diacetylphloroglucinol, have been extensively targeted in take-all-decline soils (Cook and Rovira, 1976; Weller et al., 2002; Weller et al., 2007) and soils suppressive to black root rot (Stutz et al., 1986; Laville et al., 1992; Kyselková and Moënne-Loccoz, 2012), whereas studies on soils suppressive to *R. solani* diseases have focused on *Pseudomonas* spp. producing antifungal lipopeptides (Mendes et al., 2011), *Streptomyces* spp. producing volatile metabolites (Cordovez et al., 2015) and *Paraburkholderia graminis* producing sulfurous volatile compounds (Carrión et al., 2018). In the case of soils suppressive to *Fusarium* diseases, competition with pathogenic *Fusarium* species is considered important, involving the entire soil microbiota or more specifically non-pathogenic *Fusarium* strains in Châteaurenard soils (Louvet et al., 1976; Alabouvette, 1986), or fluorescent *Pseudomonas* (iron competition; Scher and Baker, 1980; Sneh et al., 1984) in soils of Salinas Valley (California) or Châteaurenard (France). The role of extracellular lytic enzymes can be significant, as soil microbiota may protect barley from *Fusarium culmorum* via a more efficient cellulolytic activity than the pathogen, which consequently is outcompeted for nutrients (Rasmussen et al., 2002). Suppressiveness may result in part from chitinolytic effects of the soil microbiota against the pathogen, based on inhibition of *Fusarium* fungi by chitinases *in vitro* and effective protection of plant by chitinase-producing inoculants (Veliz et al., 2017). Other modes of action evidenced include the production of antifungal secondary metabolites in wilt-suppressive soils, such as a new thiopeptide by *Streptomyces* (Cha et al., 2016) and phenazines by *Pseudomonas* spp. (Mazurier et al., 2009), and immunity stimulation in banana (induction of the jasmonate and salicylic acid pathways) by fluorescent *Pseudomonas* (Lv et al., 2023).

### 5.2 Microbial diversity in soils suppressive to *Fusarium* diseases

Specific disease suppressiveness is attributed to the contribution of a few plant-benefical populations, but comparison of suppressive vs conducive soils has evidenced differences in the occurrence or prevalence of multiple taxa, in the case of suppressiveness to take all (Sanguin et al., 2009; Schreiner et al., 2010; Chng et al., 2015), black root rot (Kyselková et al., 2009), *R. solani*-mediated damping-off (Mendes et al., 2011), or potato common scab (Rosenzweig et al., 2012). Similar findings were made with soils suppressive to *Fusarium* diseases. No single phylum was uniquely associated with *F. oxysporum* wilt suppressiveness in Korean soils, even though *Actinomycetota* (formerly *Actinobacteria*) was identified as the most prevalent bacterial taxa colonizing strawberry in suppressive soils (Cha et al., 2016). Likewise, the bacterial genera *Devosia*, *Flavobacterium* and *Pseudomonas* were more abundant (and the pathogen less abundant) in Chinese soils suppressive to banana wilt than in conducive soils, and *Pseudomonas* inoculants isolated from suppressive could control the disease (Lv et al., 2023). Compared with conducive soil, Fusarium wilt suppressive soil from Châteaurenard displayed higher relative abundance of *Adhaeribacter*, *Arthrobacter, Amycolatopsis*, *Geobacter, Massilia*, *Microvirga*, *Paenibacillus*, *Rhizobium*, *Rhizobacter*, *Rubrobacter* and *Stenotrophomonas* (but not *Pseudomonas*) (Siegel-Hertz et al., 2018). However, differences were also found in the fungal community, with several fungal genera (*Acremonium*, *Ceratobasidium*, *Chaetomium*, *Cladosporium*, *Clonostachys*, *Mortierella*, *Penicillium*, *Scytalidium*, *Verticillium*, but also *Fusarium*) detected exclusively in the wilt suppressive soil (Siegel-Hertz et al., 2018). Data also pointed to a greater degree of microbial complexity in suppressive soils, with particular co-occurrence networks of taxa (Bakker et al., 2014; Lv et al., 2023). In German and Dutch soils, co-occurrence networks showed that the suppressive soil microbiota involves a guild of bacteria that probably function together, and in two of the suppressive soils this guild is dominated by *Acidobacteriota* (formerly *Acidobacteria*) (Ossowicki et al., 2020).

Many studies focused on a few, geographically-close soils, which does not provide a global view on the importance of microbial diversity. However, two studies have considered geographically diverse agricultural soils suppressive to Fusarium wilt. Various Chinese soils suppressive to banana wilt mediated by *F. oxysporum* were shown to share a common core microbiota, specific to suppressive soils, which included the genus *Pseudomonas* (Shen et al., 2022). In a wider range of soils from the Netherlands and Germany, soils suppressive to *F. culmorum*-mediated wilt of wheat did not display a specific bacterial species that correlated with suppressiveness (Ossowicki et al., 2020). There was no relation either with soil physicochemical composition (i.e. soil type, pH, contents in C, N, or bioavailable Fe, K, Mg, P) or field history, yet suppressiveness was microbial in nature, as sterilizing suppressive soils made them become conducive. This suggests that each suppressive soil may harbor its own set of phytobeneficial bacteria, supporting the notion of functional redundancy between microbiomes, meaning that different microbiomes may share common functionalities despite taxonomic differences in the microbial actors involved (Lemanceau et al., 2017). Taken together, this might be explained by the fact that protection of wheat from *F. culmorum*-mediated wilt corresponds to a case of natural suppressiveness (Ossowicki et al., 2020), where biogeographic patterns are probably important, whereas soils suppressive to Fusarium wilt of banana are induced by monoculture (Wang et al., 2019; Shen et al., 2022), with convergent effects resulting from similar banana recruitment across different soil types.

To go beyond individual analyses considered separately, we re-analyzed sequence data from five investigations comparing disease-suppressive and conducive soils of cultivated plants (flax, watermelon, bananas, and wheat) infected by different *Fusarium* species (*F. oxysporum* or *F. culmorum*). At the level of bacterial phyla, fluctuations among Châteaurenard (flax-*F. oxysporum*; Siegel-Hertz et al., 2018), Hainan (banana-*F. oxysporum*; Shen et al., 2022) (**Figure S1A**) and Dutch/German (wheat-*F. culmorum*; Ossowicki et al., 2020) (**Figure S1B**) suppressive soils were important, as were those among their conducive counterparts, and the comparison between suppressive and conducive soils at these locations was not fruitful. In another study, fluctuations among other Hainan (banana-*F. oxysporum*; Zhou et al., 2019) suppressive or conducive soils were of less magnitude, but again the comparison was not insightful (**Figure S1B**). In contrast, Jiangsu (watermelon-*F. oxysporum*; Wang et al., 2015) suppressive soils displayed a higher relative abundance of *Acidobacteriota* and *Pseudomonadota* than in conducive soils (**Figure S1B**), but this property was not relevant when considering the other locations/plant species/*Fusarium* species. Based on heatmap comparisons (**Figure S2**), the main finding was the lower prevalence of the *Bacillota* phylum in the Jiangsu (watermelon-*F. oxysporum*) suppressive vs conducive soils, which was restricted to the case of these soils.

**Figure 4:**
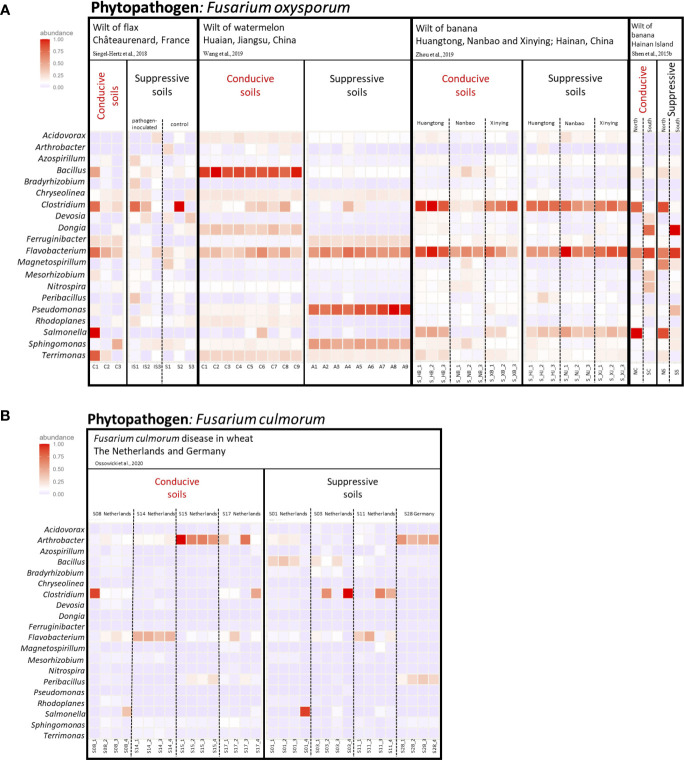
Heatmap of the major bacterial genera detected in the rhizosphere of plants grown in soils suppressive or conducive to different *Fusarium* diseases, based on analysis (**File S1**) of selected studies (Shen et al., 2015b; Siegel-Hertz et al., 2018; Wang et al., 2019; Zhou et al., 2019; Ossowicki et al., 2020). **(A)** The 20 most abundant genera in soils conducive or suppressive to diseases caused by *Fusarium oxysporum*. In Siegel-Hertz et al. (2018), suppressive soils were assessed after *Fusarium* inoculation or without. **(B)** The 20 most abundant genera in soils conducive or suppressive to diseases caused by *Fusarium culmorum*. The color intensity in each cell indicates the relative abundance (%) of a genus in each study for each plant type. When relevant, dotted lines are used to separate pathogen-inoculated samples from non-inoculated samples (in Châteaurenard) or samples from different fields. More details on individual conditions are available in **Table S2**.

At the level of bacterial genera, the comparison of Châteaurenard (flax-*F. oxysporum*), Hainan (banana-*F. oxysporum*) or Dutch/German (wheat-*F. culmorum*) soils did not lead to the identification of indicator taxa (**Figures 4**, **S3**), but at Jiangsu (watermelon-*F. oxysporum*) the genera *Bacillus*, *Dongia*, *Rhodoplanes* and *Terrimonas* were less prevalent and the genera *Ferruginibacter, Flavobacterium, Pseudomonas* and *Sphingomonas* more prevalent in suppressive soils than in conducive soils (**Figure S3A**). Therefore, the comparison between suppressive and conducive soils was sometimes meaningful at the local scale, but typically not when considering a wider range of geographic or biological (plant and *Fusarium* species) conditions together. In other words, the information available so far points that suppressiveness to *Fusarium* diseases relies on microbial selection processes by roots that depend on local conditions, i.e. probably related to microbial biogeography, soil type, plant species, *Fusarium* genotype and most likely other local factors as well.

## 6 Variability and management of soil suppressiveness to *Fusarium* diseases

### 6.1 Environmental factors influencing soil suppressiveness to *Fusarium* diseases

Environmental conditions in soil may influence *Fusarium* autecology, the composition and activity of the soil microbial community, the tripartite interactions between this microbiota, *Fusarium* pathogens and the plant, and ultimately the level of disease suppressiveness (Marshall and Alexander, 1960; Amir and Alabouvette, 1993; Mazzola, 2002; Czembor et al., 2015). Key environmental factors in this regard include soil physicochemical properties and weather conditions (Weber and Kita, 2010).

Early work on the suppressiveness of soils to vascular Fusarium diseases drew attention to the positive role of certain abiotic factors and, in particular, montmorillonite-type clays (Stover, 1956; Stotzky and Torrence Martin, 1963). In addition, higher clay contents may contribute to reduced infestation by *Fusarium* (Kurek and Jaroszuk-Ściseł, 2003; Deltour et al., 2017), by altering oxygen diffusion, pH buffering and nutrient availability (Orr and Nelson, 2018). Höper et al. (1995) showed that the level of suppressiveness to Fusarium wilt of flax increased in soils amended with montmorillonite, kaolinite or illite at pH 7. A negative correlation between soil pH and *Fusarium* disease severity was reported in experiments with flax (Senechkin et al., 2014), strawberry (Fang et al., 2012) and banana (Shen et al., 2015a). However, the correlation between pH and Fusarium wilt incidence was positive in studies on banana (Peng et al., 1999) and watermelon (Cao et al., 2016). Certain experiments acidified soil originally at pH 8.0 (Peng et al., 1999) or 7.4 (Cao et al., 2016), whereas others limed an acidic soil (Fang et al., 2012; Senechkin et al., 2014; Shen et al., 2015a). Inconsistencies may relate to the complexity of pH effects on *Fusarium* pathogens and diseases, and possible interactions with soil properties, *Fusarium* and plant genotypes, or other experimental conditions. In addition, soil suppressiveness to Fusarium wilt necessitates sufficient levels of nitrogen, as disease incidence negatively correlates with the NH_4_^+^ and NO_3_^–^ contents in the soil (Li et al., 2016; Meng et al., 2019). Moreover, the addition of calcium to the soils suppressed Fusarium wilt in several soil type × plant conditions (Spiegel et al., 1987; Peng et al., 1999; Gatch and du Toit, 2017). In Brittany, *F. graminearum* growth positively correlated with manganese and iron contents in the soil (Legrand et al., 2019). A positive correlation was found between hemicellulose concentration and suppression of Fusarium wilt in tomato and carnation (Castaño et al., 2011), as well as cellulose concentration and suppression of Fusarium seedling blight of barley (Rasmussen et al., 2002). This is attributed to the activity of cellulolytic microorganisms that limit *Fusarium* growth, as lower organic matter content (following decomposition) would reduce resources supporting this microbiota and disease suppression (Orr and Nelson, 2018).

Climatic conditions, notably temperature and precipitation may strongly affect the incidence of *Fusarium* diseases (Orr and Nelson, 2018). Phytopathogenic species *F. oxysporum*, *F. solani*, *F. verticillioides*, *F. graminearum* and *F. culmorum* develop best under humid conditions, at water activity above 0.86 (**Table S1**) (Thrane, 2014). Severity of Fusarium wilt in lettuce (Scott et al., 2009; Ferrocino et al., 2013) and FHB in wheat was positively correlated with soil temperature (Xu et al., 2007; Nazari et al., 2018). For example, Fusarium wilt incidence significantly increased when lettuce was grown at 22-26°C instead of 18-22°C (Ferrocino et al., 2013). Similarly, in both conducive and suppressive soils, severity of Fusarium wilt of banana was significantly increased when temperature was raised from 24°C to 34°C (Peng et al., 1999).

### 6.2 Farming practices and the management of soil suppressiveness to *Fusarium* diseases

As many other soil-inhabiting pathogenic fungi, the *Fusarium* spp. can overwinter as mycelium in plant debris or dormant structures in the soil, which leads to cause the initial infection of plants in the following season (Nelson et al., 1994; Janvier et al., 2007; Leplat et al., 2013; Xu et al., 2021). Therefore, cultural practices removing the primary inoculum of the pathogen from overwintering soils are useful to prevent future infection (Voigt, 2002). However, farming practices also influence soil suppressiveness by shaping the rhizosphere microbial community (Campos et al., 2016) and stimulating the activity of beneficial rhizosphere microorganisms (Janvier et al., 2007). In this context, various agricultural practices, such as crop rotation/monocropping, tillage, organic amendments and fertilisers, are important to consider to develop suppressiveness-based control methods in farm fields (Janvier et al., 2007; Fu et al., 2016).

Except in the few cases where monoculture induces suppressiveness to *Fusarium* diseases (Larkin et al., 1993; Shen et al., 2022), cropping systems based on rotation of different plant species result in reduced survival of soil-borne pathogen propagules over the short term (Winter et al., 2014). Crop rotation may reduce severity and incidence of diseases caused by *Fusarium* spp. (Wang et al., 2015; Khemir et al., 2020). For example, compared with the tomato monoculture, soil management under wheat - tomato rotation changes soil microbial composition by increasing the abundance of microbial taxa such as *Bacillus*, *Paenibacillus*, *Pseudomonas*, *Streptomyces*, *Aspergillus*, *Penicillium* and *Mortierella*, which may control Fusarium wilt of tomato (De Corato et al., 2020). Reduced incidence of *F. pseudograminearum* and *F. culmorum* in the soils under cereal – legumes rotation management may be due to the non-host character of the legumes (Evans et al., 2010). However, not all crop rotations lead to reduced disease pressure (Ranzi et al., 2017). In the case of the FHB, it was advocated to rotate wheat and corn with crops like soybean, until it was shown that *F. graminearum* can also cause disease in soybean, as it has a wide range of hosts (Marburger et al., 2015). This suggests that there is no common rule regarding the relationship between crop rotation and *Fusarium* disease incidence.

Crop residues of high cellulose content promoted the activity of beneficial cellulolytic microorganisms and limited the development of *Fusarium culmorum* (Rasmussen et al., 2002), as organic amendments represent a favorable environment for beneficial microorganisms that are able to combat phytopathogenic *Fusarium* species (Maher et al., 2008; Cuesta et al., 2012). Accordingly, organic amendments like animal manure, solid wastes and different composts are often used to improve soil health by delivering nutrients to the soil and also by stimulating beneficial microbiota (Fu et al., 2016; Mousa and Raizada, 2016). Thus, soils with added organic amendments exhibited inhibitory effects against *F. verticillioides* by reducing the production of a fungal pigment and sporulation, consequently disabling fungal spread (Nguyen et al., 2018). Addition of vermicompost reduced tomato infection by *F. oxysporum* f. sp. *lycopersici* (Szczech, 1999) and mulched straw contributed to the suppression of seedling blight caused by *F. culmorum* (Knudsen et al., 1999). Soils supplemented with coffee residue compost or rapeseed meal exhibited suppressiveness to *F. oxysporum*-mediated wilt, and microorganisms isolated from supplemented soils inhibited *F. oxysporum* growth on agar plates (Mitsuboshi et al., 2018). Carbon addition to soil influenced the soil microbiome, enhancing *Fusarium*-inhibitory populations from the *Streptomyces* genus (Dundore-Arias et al., 2020). However, increasing organic matter content may promote *Fusarium* survival in certain (rare) cases. One study tested the effects of 18 composts (made from different mixtures of manure, domestic biowaste and green waste) on Fusarium wilt disease suppression, caused by *F. oxysporum* f. sp. *lini*, and it was shown that only one compost did not positively affect the disease suppression (Termorshuizen et al., 2006). The efficiency of organic amendments in controlling plant diseases is determined by the pathosystem, the application rate, the kind of amendment and the level of maturity of composts or disintegration phase of crop residues (Janvier et al., 2007).

Tillage, which is one factor influencing organic matter decomposition, appears to have contrasting effects on soil suppressiveness. Under conventional tillage, tillage depth appears to play a crucial role in soil survival of *Fusarium*, such that the deeper the tillage, the lower the abundance of *Fusarium* species (Steinkellner and Langer, 2004). This can be partly explained by the fact that the pathogen is displaced from its niche, reducing its ability to survive (Bailey and Lazarovits, 2003), and the rate of decomposition of buried residues is faster than at the soil surface (Leplat et al., 2013). The carbon released during these decomposition processes increases the activity of the soil microbiota, thereby improving the overall functioning of the soil (Bailey and Lazarovits, 2003). Under conservation tillage, surface residues persist and can act as a long-term source of inoculum for plant infection by *F. verticillioides*, *F. proliferatum* and *F. subglutinans*, as they can colonise crop residues and produce overwintering spores that often survive the period when plants are absent from the agrosystem (Bockus and Shroyer, 1998; Cotten and Munkvold, 1998; Pereyra et al., 2004). This is consistent with results suggesting that conservation tillage and leaving crop residues *in situ* increase *Fusarium* abundance (Govaerts et al., 2008; Wang et al., 2020). For example, spores of *Fusarium* species could be recovered from plant residues more than two years after harvest (Pereyra et al., 2004). In certain cases, lower occurrence of plant infection by *F. culmorum*, *F. equiseti* (Weber et al., 2001) and *F. pseudograminearum* (Theron et al., 2023) was found under conservation tillage compared with conventional tillage. These contrasting results might be due to differences in environmental factors, cropping patterns and soil types, which could modulate interactions between soil conditions, *Fusarium* ecology and plant physiology (Sturz and Carter, 1995). The use of simplified tillage practices was proposed to reduce *F. culmorum* abundance, by mixing crop residues with the topsoil layer to promote the growth of beneficial straw-decomposing microorganisms (Weber and Kita, 2010).

Different fertilizers have different effects on phytopathogenic *Fusarium* spp. On one hand, the development of FHB caused by *F. culmorum* and *F. graminearum* increased with inorganic nitrogen fertilization (Lemmens et al., 2004), and on the other hand, nitrite could reduce the population of *F. oxysporum* (Löffler et al., 1986). Besides, higher doses of nitrogen may contribute to higher accumulation of *Fusarium* mycotoxins (Podolska et al., 2017). The addition of phosphorus fertilizer, in the form of P_2_O_5_, significantly reduced *Fusarium*-caused wilting in chickpea, lentil and lupine, in both greenhouse and field conditions (Elhassan et al., 2010). Organic fertilizers can lead to an increase in indigenous microbial populations, thus contributing to suppression of Fusarium wilt disease (Montalba et al., 2010; Raza et al., 2015). When grown with the addition of organic N fertilizer, highbush blueberry exhibited increased tolerance to *F. solani*, in parallel to increased soil microbial activity and mycorrhizal colonization (Montalba et al., 2010).

## 7 Conclusion and outlook

Disease-suppressiveness of soils is a useful model to understand microbial phytoprotection and develop sustainable plant protection strategies for soils devoid of this property. In this review, we summarized the current knowledge on *Fusarium* phytopathogens, the available control methods and soils suppressive to *Fusarium* diseases, with the underlying mechanisms involved in the suppression. On one hand, extensive information is available on environmental and microbial properties responsible for suppressiveness to *Fusarium* diseases. One prominent feature is the diversity of *Fusarium*-based pathosystems for which suppressive soils are documented, in terms of *Fusarium* species (often *F. oxysporum*, but not only), host plants (both monocots and dicots), types of disease (often wilt, but not only), geographic locations of soil and farming conditions, and types of suppressiveness (i.e. natural suppressiveness to *Fusarium* diseases, but also monoculture-induced suppressiveness as well as fungistasis towards *Fusarium* pathogens). This diversity is paralleled by differences in microbiota composition and diversity associated with disease control in the different cases of suppressiveness. On the other hand, despite the fact that soils suppressive to *Fusarium* diseases have been studied for decades, they are still poorly understood in terms of microbiota functioning, and knowledge remains fragmented.

On this basis, additional research is needed to integrate further the scientific approaches used to decipher suppressiveness to *Fusarium* diseases. First, by combining complementary assessment methodology with current next-generation sequencing and ecological networks research, and incorporating experimental strategies to manipulate and transplant rhizosphere microbiome (or single microorganisms) of plants grown in suppressive soils to those in conducive soils to go beyond correlative work, as started recently (Ye et al., 2020; Jiang et al., 2022). Second, by extending the range of soil conditions investigated, and develop meta-analyses to estimate key microbiota differences between suppressive and conducive soils, as pioneered by Yuan et al. (2020). Third, by considering a wider range of biological actors, including beneficial fungi (often neglected), soil fauna (likely to influence microbial communities, *Fusarium* vectorisation, and plant health; e.g. Dita et al., 2018; Wagner et al., 2022). Fourth, by taking into account plant genetics, behavior and physiological responses to *Fusarium* pathogens (e.g. Liu et al., 2019). Therefore, there is a need for a more multidisciplinary approach to understand microbiota functioning in soils suppressive to *Fusarium* diseases.

## Author contributions

All authors contributed to the writing of this review article and approved the submitted version.
