# Supplementary material for: Microbial diversity in soils suppressive to Fusarium diseases
Source: Front Plant Sci. 2023 Dec 4;14:1228749. doi: 10.3389/fpls.2023.1228749 (PMC10726057; doi:10.3389/fpls.2023.1228749)
Supplement: Supplementary file 1 [file DataSheet_1.docx]

**Supplementary File**

**Microbial diversity in soils suppressive to *Fusarium* diseases**

Irena Todorović^1,2^, Yvan Moënne-Loccoz^1^, Vera Raičević^2^, Jelena Jovičić-Petrović^2^ and Daniel Muller^1*^

^1^Univ Lyon, Université Claude Bernard Lyon 1, CNRS, INRAE, VetAgro Sup, UMR5557 Ecologie Microbienne, 43 bd du 11 novembre 1918, F-69622 Villeurbanne, France

^2^University of Belgrade, Faculty of Agriculture, Nemanjina 6, 11080 Zemun, Belgrade, Serbia

***Correspondence: Daniel Muller, daniel.muller@univ-lyon1.fr**

**
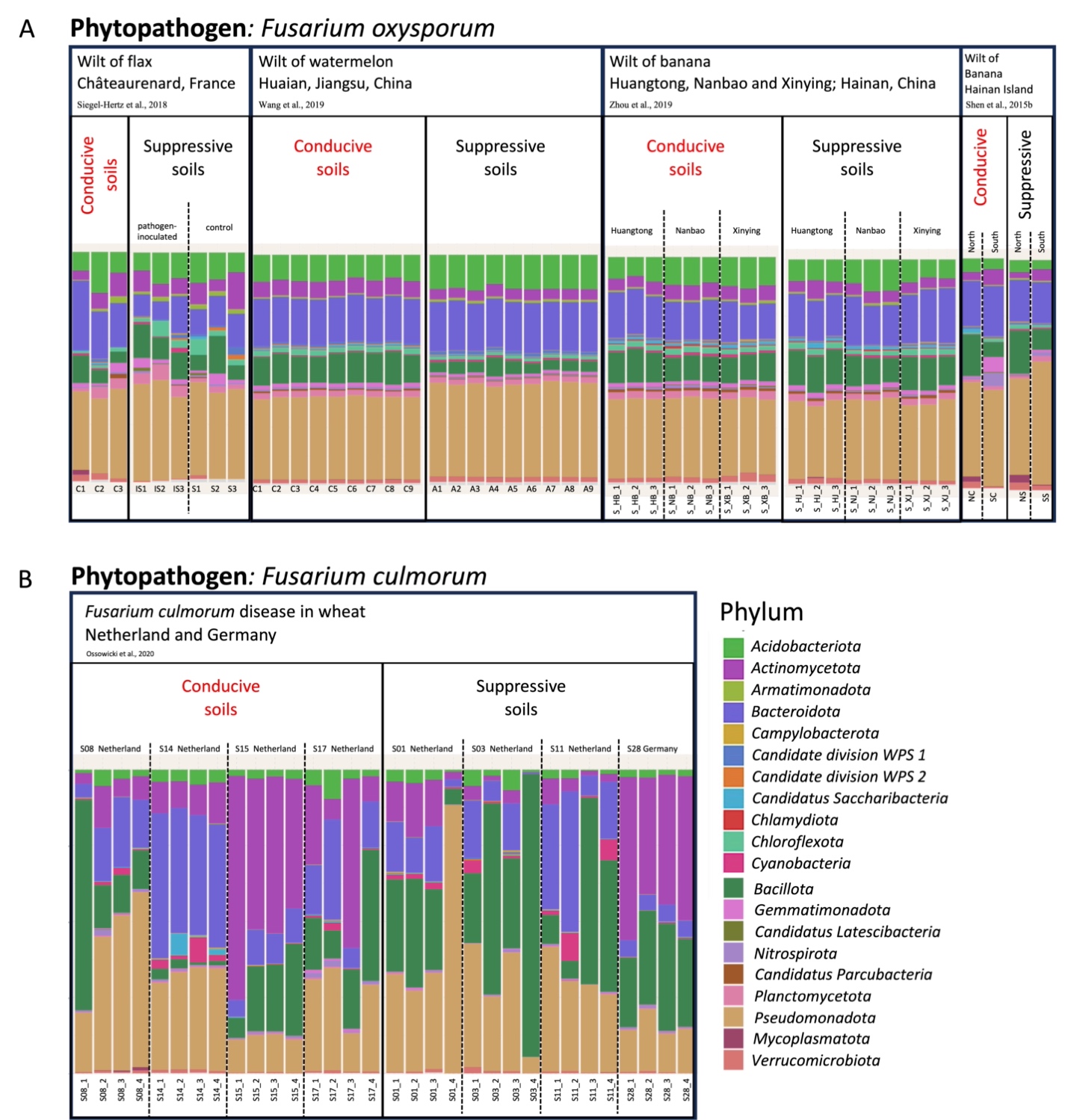
**

**Supplementary figure 1.** Stacked barplot of the bacterial phyla detected in the rhizosphere of plants grown in soils suppressive or conducive to different *Fusarium* diseases, based on analysis (File S1) of selected studies (Shen et al., 2015b; Siegel-Hertz et al., 2018; Wang et al., 2019; Zhou et al., 2019; Ossowicki et al., 2020). **(A)** *Fusarium oxysporum*-conducive or suppressive soils. In Siegel-Hertz et al. (2018), suppressive soils were assessed after *Fusarium* inoculation or before. **(B)** *Fusarium culmorum*-conducive or suppressive soils. When relevant, dotted lines are used to separate pathogen-inoculated samples from non-inoculated samples (in Châteaurenard) or samples from different fields. More details on individual conditions are available in Table S2.

**
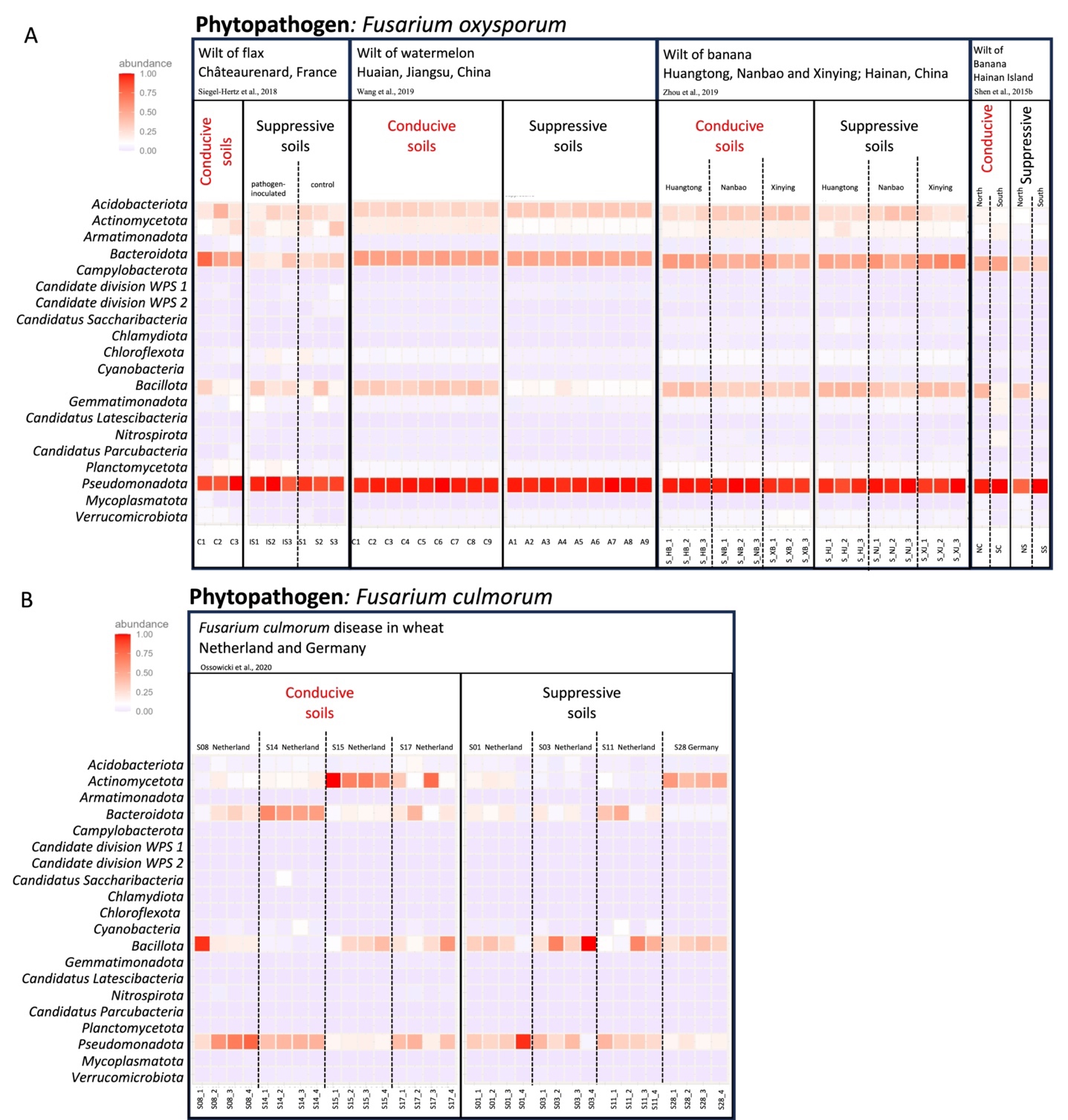
Supplementary figure 2.** Heatmap of the bacterial phyla detected in the rhizosphere of plants grown in soils suppressive or conducive to different *Fusarium* diseases, based on analysis (File S1) of selected studies (Shen et al., 2015b; Siegel-Hertz et al., 2018; Wang et al., 2019; Zhou et al., 2019; Ossowicki et al., 2020). (**A**) The 20 most abundant phyla in soils conducive or suppressive to diseases caused by *Fusarium oxysporum*. In Siegel-Hertz et al. (2018), suppressive soils were assessed after *Fusarium* inoculation or before. (**B**) The 20 most abundant phyla in soils conducive or suppressive to diseases caused by *Fusarium culmorum*. The color intensity in each cell indicates the relative abundance (%) of a phylum in each study for each plant type. When relevant, dotted lines are used to separate pathogen-inoculated samples from non-inoculated samples (in Châteaurenard) or samples from different fields. More details on individual conditions are available in Table S2.

**
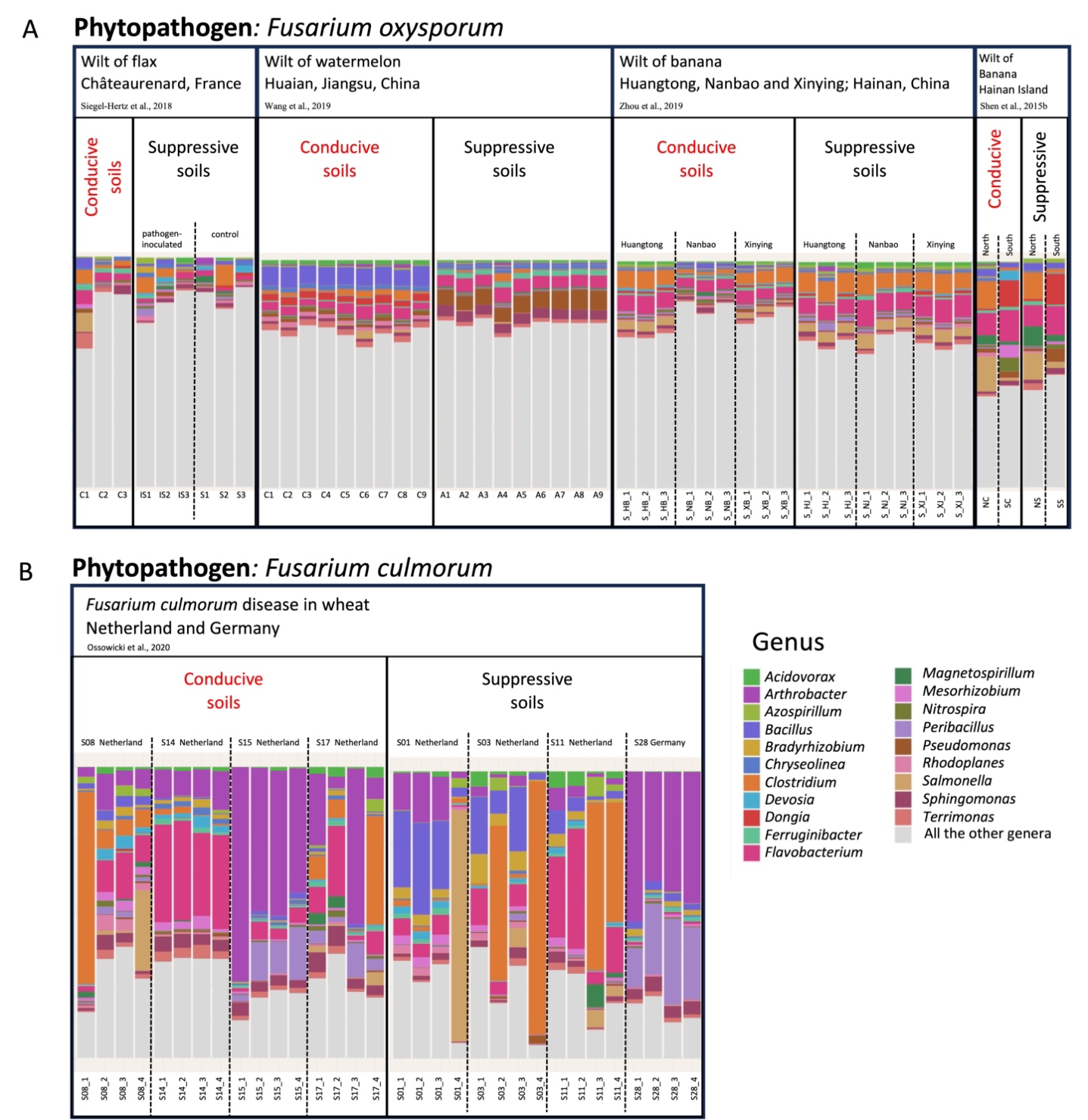
 Supplementary figure 3.** Stacked barplot of the 20 major bacterial genera detected in the rhizosphere of plants grown in soils suppressive or conducive to different *Fusarium* diseases, based on analysis (File S1) of selected studies (Shen et al., 2015b; Siegel-Hertz et al., 2018; Wang et al., 2019; Zhou et al., 2019; Ossowicki et al., 2020). **(A)** *Fusarium oxysporum*-conducive or suppressive soils. In Siegel-Hertz et al. (2018), suppressive soils were assessed after *Fusarium* inoculation or before. **(B)** *Fusarium culmorum*-conducive or suppressive soils. When relevant, dotted lines are used to separate pathogen-inoculated samples from non-inoculated samples (in Châteaurenard) or samples from different fields. More details on individual conditions are available in Table S2.

**Table S1.** *Fusarium* species and optimum temperature, pH and water activity for growth

| Species | Optimum temperature | Optimum pH | Optimum a_w_ | Reference |
| --- | --- | --- | --- | --- |
| *F. oxysporum* | 25 to 27.5 ºC | 5.1 to 5.9 | > 0.89 | Thrane, 2014; Jiménez-Díaz et al., 2015 |
| *F. solani* | ~ 29 ºC | ~ 7.5 | > 0.90 | Thrane, 2014; Mohsen et al., 2015; Yan and Nelson, 2020 |
| *F. verticillioides* | ~ 27 ºC | 6 to 7 | > 0.87 | Thrane, 2014; Kumar et al., 2019 |
| *F. graminearum* | 25 to 30 ºC | ~ 3.5 | > 0.90 | Thrane, 2014; Panwar et al., 2017 |
| *F. culmorum* | ~ 25 ºC | ~ 5 | > 0.87 | Aleandri et al., 2007; Scherm et al., 2013; Thrane, 2014 |

| **Table S2.** Published data used for comparative analysis of bacterial communities in rhizosphere soil of plants cultivated in soils conducive or suppressive to *Fusarium* diseases. | | | | | |
| --- | --- | --- | --- | --- | --- |
| File name | Soil status | sSample | Location | Plant | References |
| conducive_S08_1 | conducive | S08_1 | S08 Netherland | wheat | Ossowicki, A., Tracanna, V., Petrus, M. L. C., van Wezel, G., Raaijmakers, J. M., Medema, M. H., et al. (2020). Microbial and volatile profiling of soils suppressive to *Fusarium culmorum* of wheat. *Proc. Biol. Sci.* 287, 20192527. doi: 10.1098/rspb.2019.2527. |
| conducive_S08_2 | conducive | S08_2 | S08 Netherland | wheat |  |
| conducive_S08_3 | conducive | S08_3 | S08 Netherland | wheat |  |
| conducive_S08_4 | conducive | S08_4 | S08 Netherland | wheat |  |
| conducive_S14_1 | conducive | S14_1 | S14 Netherland | wheat |  |
| conducive_S14_2 | conducive | S14_2 | S14 Netherland | wheat |  |
| conducive_S14_3 | conducive | S14_3 | S14 Netherland | wheat |  |
| conducive_S14_4 | conducive | S14_4 | S14 Netherland | wheat |  |
| conducive_S15_1 | conducive | S15_1 | S15 Netherland | wheat |  |
| conducive_S15_2 | conducive | S15_2 | S15 Netherland | wheat |  |
| conducive_S15_3 | conducive | S15_3 | S15 Netherland | wheat |  |
| conducive_S15_4 | conducive | S15_4 | S15 Netherland | wheat |  |
| conducive_S17_1 | conducive | S17_1 | S17 Netherland | wheat |  |
| conducive_S17_2 | conducive | S17_2 | S17 Netherland | wheat |  |
| conducive_S17_3 | conducive | S17_3 | S17 Netherland | wheat |  |
| conducive_S17_4 | conducive | S17_4 | S17 Netherland | wheat |  |
| suppressive_S01_1 | suppressive | S01_1 | S01 Netherland | wheat |  |
| suppressive_S01_2 | suppressive | S01_2 | S01 Netherland | wheat |  |
| suppressive_S01_3 | suppressive | S01_3 | S01 Netherland | wheat |  |
| suppressive_S01_4 | suppressive | S01_4 | S01 Netherland | wheat |  |
| suppressive_S03_1 | suppressive | S03_1 | S03 Netherland | wheat |  |
| suppressive_S03_2 | suppressive | S03_2 | S03 Netherland | wheat |  |
| suppressive_S03_3 | suppressive | S03_3 | S03 Netherland | wheat |  |
| suppressive_S03_4 | suppressive | S03_4 | S03 Netherland | wheat |  |
| suppressive_S11_1 | suppressive | S11_1 | S11 Netherland | wheat |  |
| suppressive_S11_2 | suppressive | S11_2 | S11 Netherland | wheat |  |
| suppressive_S11_3 | suppressive | S11_3 | S11 Netherland | wheat |  |
| suppressive_S11_4 | suppressive | S11_4 | S11 Netherland | wheat |  |
| suppressive_S28_1 | suppressive | S28_1 | S28 Germany | wheat |  |
| suppressive_S28_2 | suppressive | S28_2 | S28 Germany | wheat |  |
| suppressive_S28_3 | suppressive | S28_3 | S28 Germany | wheat |  |
| suppressive_S28_4 | suppressive | S28_4 | S28 Germany | wheat |  |
| DRR018526_conducive_NC1 | conducive | NC1 | North Hainan Island, China | banana | Shen, Z., Ruan, Y., Xue, C., Zhong, S., Li, R., and Shen, Q. (2015b). Soils naturally suppressive to banana Fusarium wilt disease harbor unique bacterial communities. *Plant Soil.* 393, 21–33. doi: 10.1007/s11104-015-2474-9 |
| DRR018526_conducive_NC2 | conducive | NC2 | North Hainan Island, China | banana |  |
| DRR018526_conducive_NC3 | conducive | NC3 | North Hainan Island, China | banana |  |
| DRR018526_conducive_SC1 | conducive | SC1 | South Hainan Island, China | banana |  |
| DRR018526_conducive_SC2 | conducive | SC2 | South Hainan Island, China | banana |  |
| DRR018526_conducive_SC3 | conducive | SC3 | South Hainan Island, China | banana |  |
| DRR018526_suppressive_NS1 | suppressive | NS1 | North Hainan Island, China | banana |  |
| DRR018526_suppressive_NS2 | suppressive | NS2 | North Hainan Island, China | banana |  |
| DRR018526_suppressive_NS3 | suppressive | NS3 | North Hainan Island, China | banana |  |
| DRR018526_suppressive_SS1 | suppressive | SS1 | South Hainan Island, China | banana |  |
| DRR018526_suppressive_SS2 | suppressive | SS2 | South Hainan Island, China | banana |  |
| DRR018526_suppressive_SS3 | suppressive | SS3 | South Hainan Island, China | banana |  |
| C1_16S_conducive | conducive | C1 | Châteaurenard, France | flax | Siegel-Hertz, K., Edel-Hermann, V., Chapelle, E., Terrat, S., Raaijmakers, J. M., and Steinberg, C. (2018). Comparative microbiome analysis of a Fusarium wilt suppressive soil and a Fusarium wilt conducive soil from the Châteaurenard region. *Front. Microbiol.* 9, 568. |
| C2_16S_conducive | conducive | C2 | Châteaurenard, France | flax |  |
| C3_16S_conducive | conducive | C3 | Châteaurenard, France | flax |  |
| IS1_16S_suppressive | suppressive | IS1 | Châteaurenard, France inoculated with Fusarium | flax |  |
| IS2_16S_suppressive | suppressive | IS2 | Châteaurenard, France inoculated with Fusarium | flax |  |
| IS3_16S_suppressive | suppressive | IS3 | Châteaurenard, France inoculated with Fusarium | flax |  |
| S1_16S_suppressive | suppressive | S1 | Châteaurenard, France | flax |  |
| S2_16S_suppressive | suppressive | S2 | Châteaurenard, France | flax |  |
| S3_16S_suppressive | suppressive | S3 | Châteaurenard, France | flax |  |
| SRR7415810_A1 | suppressive | A1 | Huaian, Jiangsu, China | watermelon | Wang, T., Hao, Y., Zhu, M., Yu, S., Ran, W., Xue, C., ... and Shen, Q. (2019). Characterizing differences in microbial community composition and function between Fusarium wilt diseased and healthy soils under watermelon cultivation. *Plant Soil*, 438, 421-433. https://doi.org/10.1007/s11104-019-04037-6 |
| SRR7415809_A2 | suppressive | A2 | Huaian, Jiangsu, China | watermelon |  |
| SRR7415804_A3 | suppressive | A3 | Huaian, Jiangsu, China | watermelon |  |
| SRR7415801_A4 | suppressive | A4 | Huaian, Jiangsu, China | watermelon |  |
| SRR7415802_A5 | suppressive | A5 | Huaian, Jiangsu, China | watermelon |  |
| SRR7415800_A6 | suppressive | A6 | Huaian, Jiangsu, China | watermelon |  |
| SRR7415803_A7 | suppressive | A7 | Huaian, Jiangsu, China | watermelon |  |
| SRR7415798_A8 | suppressive | A8 | Huaian, Jiangsu, China | watermelon |  |
| SRR7415815_A9 | suppressive | A9 | Huaian, Jiangsu, China | watermelon |  |
| SRR7415813_C1 | conducive | C1 | Huaian, Jiangsu, China | watermelon |  |
| SRR7415812_C2 | conducive | C2 | Huaian, Jiangsu, China | watermelon |  |
| SRR7415811_C3 | conducive | C3 | Huaian, Jiangsu, China | watermelon |  |
| SRR7415799_C4 | conducive | C4 | Huaian, Jiangsu, China | watermelon |  |
| SRR7415814_C5 | conducive | C5 | Huaian, Jiangsu, China | watermelon |  |
| SRR7415808_C6 | conducive | C6 | Huaian, Jiangsu, China | watermelon |  |
| SRR7415807_C7 | conducive | C7 | Huaian, Jiangsu, China | watermelon |  |
| SRR7415806_C8 | conducive | C8 | Huaian, Jiangsu, China | watermelon |  |
| SRR7415805_C9 | conducive | C9 | Huaian, Jiangsu, China | watermelon |  |
| SRR6701374_diseased_HB_2_bacteria | conducive | C_HB_2 | from the Huangtong farm, Hainan, China | banana | Zhou, D., Jing, T., Chen, Y., Wang, F., Qi, D., Feng, R., ... and Li, H. (2019). Deciphering microbial diversity associated with Fusarium wilt- diseased and disease-free banana rhizosphere soil. *BMC Microbiol.* 19, 1-13. https://doi.org/10.1186/s12866-019-1531-6 |
| SRR6701375_diseased_HB_1_bacteria | conducive | C_HB_1 | from the Huangtong farm, Hainan, China | banana |  |
| SRR6701377_diseased_HB_3_bacteria | conducive | C_HB_3 | from the Huangtong farm, Hainan, China | banana |  |
| SRR6701387_diseased_XB_3_bacteria | conducive | C_XB_3 | from the Xinying farm, Hainan, China | banana |  |
| SRR6701389_diseased_NB_3_bacteria | conducive | C_NB_3 | from the Nanbao farm, Hainan, China | banana |  |
| SRR6701390_diseased_NB_2_bacteria | conducive | C_NB_2 | from the Nanbao farm, Hainan, China | banana |  |
| SRR6701391_diseased_NB_1_bacteria | conducive | C_NB_1 | from the Nanbao farm, Hainan, China | banana |  |
| SRR6701392_diseased_XB_2_bacteria | conducive | C_XB_2 | from the Xinying farm, Hainan, China | banana |  |
| SRR6701393_diseased_XB_1_bacteria | conducive | C_XB_1 | from the Xinying farm, Hainan, China | banana |  |
| SRR6701372_healthy_XJ_3_bacteria | suppressive | S_XJ_3 | from the Xinying farm, Hainan, China | banana |  |
| SRR6701373_healthy_XJ_2_bacteria | suppressive | S_XJ_2 | from the Xinying farm, Hainan, China | banana |  |
| SRR6701376_healthy_HJ_1_bacteria | suppressive | S_HJ_1 | from the Huangtong farm, Hainan, China | banana |  |
| SRR6701378_healthy_HJ_3_bacteria | suppressive | S_HJ_3 | from the Huangtong farm, Hainan, China | banana |  |
| SRR6701379_healthy_HJ_2_bacteria | suppressive | S_HJ_2 | from the Huangtong farm, Hainan, China | banana |  |
| SRR6701386_healthy_XJ_1_bacteria | suppressive | S_XJ_1 | from the Xinying farm, Hainan, China | banana |  |
| SRR6701388_healthy_NJ_1_bacteria | suppressive | S_NJ_1 | from the Nanbao farm, Hainan, China | banana |  |
| SRR6701394_healthy_NJ_3_bacteria | suppressive | S_NJ_3 | from the Nanbao farm, Hainan, China | banana |  |
| SRR6701395_healthy_NJ_2_bacteria | suppressive | S_NJ_2 | from the Nanbao farm, Hainan, China | banana |  |

**File S1.** Supplementary methods for comparing community composition.

We selected 5 studies for re-analysis based on two criteria: the presence of studies on soils emblematic of resistance to fusariosis, such as that of Châteaurenard, and on different tested pathosystems. The second criterion was based on the accessibility of data in databases; some studies were excluded because it was impossible to infer the samples. All the data from selected studies were downloaded using their accession numbers from the SRA, NCBI, or EBI websites. Read quality was assessed, and primers were removed using the software fastp (version 0.23.2) with default parameters. Subsequently, all reads underwent processing using vsearch software (version 2.23.0) following the classical workflow steps: filtering, trimming, dereplicating, clustering, chimera removal, and final assignment. Taxonomy was determined using the sintax algorithm implemented in the vsearch software against the RDP sequence database training set No.18. Statistical analyses were performed using R, primarily with the phyloseq package (version 1.42.0), vegan package (version 2.6-4), and ade4 package (version 1.7-22). Graphs and figures were generated using the ggplot2 package (version 3.4.1).

We utilized samples from the following sources: Wang et al., 2019 (Accession number: SRP151181), Shen et al., 2015b (Accession number: DRA002235), Zhou et al., 2019 (Accession number: SRP132524), and Ossowicki et al. 2020 (Accession number: PRJEB24081). Additionally, data from Ossowicki et al., 2020, are accessible through the Dryad Digital Repository: <https://dx.doi.org/10.5061/dryad.g884q70>.
